# Design and Evaluation of a Macroarray for Detection, Identification, and Typing of Viral Hemorrhagic Septicemia Virus (VHSV)

**DOI:** 10.3390/ani11030841

**Published:** 2021-03-16

**Authors:** Carmen López-Vázquez, Isabel Bandín, Carlos P. Dopazo

**Affiliations:** Unidad de Ictiopatología, Instituto de Acuicultura y Departamento de Microbiología, Universidad de Santiago de Compostela, 15782 Santiago de Compostela, Spain; mdelcarmen.lopez.vazquez@usc.es (C.L.-V.); isabel.bandin@usc.es (I.B.)

**Keywords:** diagnosis, viral haemorrhagic septicaemia virus

## Abstract

**Simple Summary:**

In the present report will be the first of a series of PCR-based macroarrays for the detection, typing, and quantification of several viruses of importance in fish aquaculture. The way of preparing the array—under patent process—differs from macroarrays reported by other researchers because it ensures its reliability after storage times up to 1 year at −25 °C. In this study, we show the results of the evaluation of a macroarray designed for the viral hemorrhagic septicemia virus (VHSV), of great importance for fish farmers worldwide, and the same procedure of validation is ongoing with macroarrays for other fish viruses.

**Abstract:**

The viral hemorrhagic septicemia virus (VHSV) is the causative agent of an important disease in freshwater and marine fishes. Its diagnosis officially relies on the isolation of the virus in cell culture and its identification by serological or polymerase chain reaction (PCR) methodologies. Nowadays, reverse transcription real-time quantitative PCR (RT-qPCR) is the most widely employed technique for the detection of this virus and some studies have reported the validation of RT-qPCR procedures for the detection, typing, and quantification of VHSV isolates. However, although the efficacy of this technique is not in doubt, it can be cumbersome and even impractical when it comes to processing large numbers of samples, a situation in which cross-contamination problems cannot be ruled out. In the present study, we have designed and validated a macroarray for the simultaneous detection, typing, and quantification of VHSV strains. Its analytical sensitivity (5–50 TCID_50_/_mL_), analytical specificity (intra and intergroup), efficiency (E = 100.0–101.1) and reliability (repeatability and reproducibility with CV < 5%, and standard curves with R^2^ < 0.95) with strains from any VHSV genotype have been widely demonstrated. The procedure is based on the ‘binary multiplex RT-qPCR system (bmRT-qPCR)’ previously reported by the same team, applied to arrays of 96-well PCR strip tubes plates, which can be stored at −25 °C for three months and up to one year before their use, without significant loss of efficiency.

## 1. Introduction

Viral hemorrhagic septicemia (VHS), a disease widely distributed among freshwater and marine fishes, is one of the most serious viral diseases affecting farmed rainbow trout in Europe [1,2]. The causative agent, the viral hemorrhagic septicemia virus (VHSV), causes mortality in fish throughout their life cycle, and in many cases can produce a persistent carrier state in the host. Those carrier fish can release virus into the environment and act as a source of infection to healthy fish and other marine organisms [3].

VHSV is a member of the genus *Novirhabdovirus*, belonging to the family *Rhabdoviridae*. Based on genome sequencing, the VHSV isolates have been divided into four genogroups, named from genogroups I to IV; additionally, genogroups I and IV are divided into different sublineages [4,5,6,7,8,9]. 

The international standards to facilitate the control of the disease caused by this virus include isolation of the virus in cell culture and further identification by serological techniques or by reverse transcription PCR (RT-PCR) [10]. In addition, due to the implications that the viral genotypes—recognized by the OIE—can have on its real risk level of the disease, and due to the international norms regarding aquaculture trade [11], the typing of new isolates is becoming an additional need for diagnostic laboratories. In fact, genotyping is accepted as an important tool for tracing isolates in molecular epidemiology [12]. To this regard, our group has recently reported reverse transcription real-time quantitative PCR (RT-qPCR) procedures for diagnosis and quantification [13] and for typing [14] of this virus. Since those procedures were based on TaqMan^®^ probes, we considered the possibility of adapting them to the array technology.

Such technology was originally designed by immobilizing oligonucleotide probes to solid membranes for subsequent colorimetric detection [15]; it has created a breakthrough in diagnostics, allowing many specific sequences to be analyzed simultaneously, and has been adapted for detection of a number of viruses [16]. Arrays work in a number of different ways, but the common characteristic is that many specific pieces of nucleic acid can be identified by the use of complementary probes that make up the array [17]. However, since it is based on nucleic acid hybridization (NAH), and because the handicap of NAH is its relative low sensitivity [18], it would not be an appropriate option for diagnosis of this virus, especially in asymptomatic fish. Nevertheless, its combination with PCR amplification solves this drawback, and thus it has been widely applied in recent studies using both macro and microarrays for the diagnosis of viral diseases [19,20,21,22,23,24]. 

In the study cited above [14], we reported the validation of a binary multiplex RT-qPCR (bmRT-qPCR) for the diagnosis and typing of VHSV strains from any genotype. Therefore, we used that method to design an RT-qPCR based macroarray for the diagnosis and typing of VHSV. By using two sets of multiplex PCR and five DNA probes with three different fluorophores, we have developed a simple, robust, and rapid diagnostic platform capable of the simultaneous detection and characterization of the four genotypes of VHSV including sublineages IVa and IVb. Although the qPCR procedure the array was based on has been already validated, we thought necessary to demonstrate that the macroarray maintained all the characteristics of high analytical sensitivity, analytical specificity, and efficiency of the bmRT-qPCR method; therefore, we have carried out the present study, where we have demonstrated the reliability of the proposed macroarray.

## 2. Materials and Methods

### 2.1. Cell Culture and Virus

The epithelioma papulosum cyprinid (EPC) fish cell line (ECACC 93120820), derived from common carp (*Cyprinus carpio* L.) was maintained at 20 °C in Eagle’s minimum essential medium (EMEM; GIBCO, ThermoFisher, Madrid, Spain) containing 10% fetal bovine serum (FBS; GIBCO), penicillin (100 IU/mL) (GIBCO) and streptomycin (100 μg/mL) (GIBCO), buffered with 7.5% sodium bicarbonate (GIBCO).

Since the macroarray is based on a previously reported PCR amplification procedure validated against a large number of VHSV and non-VHSV rhabdoviruses [14], the number of VHSV strains employed in the present study was limited, focused to ensure that that reliability of the array was not affected. The reference VHSV viral strains used were FR-07-71 (Genogroup Ia; [25]), DK-1p49 (Genogroup II; [1]), MLA98/6WH1 (G. III; [26]), US-MAKAH (G. IVa; [27]), Goby 1-5 (G. IVb; [28]). The viruses were replicated in EPC cells using EMEM with 2% FBS and antibiotics as described. When the cytopathic effect became extensive, the supernatant was harvested and centrifuged to eliminate cell debris. Clarified supernatants were then used for the subsequent experiments.

### 2.2. Viral Titration

The viral titrations were performed using the endpoint dilution method, as previously described [29], using 96-well plates with EPC cells (3 replicas per dilution), and the titers determined by the Reed and Müench [30] procedure, and given as TCID_50_/_mL_.

### 2.3. Primers and Probes

The PCR amplification step of this macroarray is on in the binary multiplex RT-qPCR (bmRT-qPCR) for the diagnosis and typing of VHSV strains of the 4 genogroups, designed and validated by Vázquez et al. [14]. In this procedure, the system consists of 2 PCR tubes, MSI and MSII (multiplex systems I and II). MSI uses a set of 2 primers and 3 probes designed for the European genotypes; the probes are specific for genotypes I (FAM labelled), II (HEX labelled) and III (Tex Red labelled). MSII uses 2 sets of 2 primers and 1 probe; one is specific for genogroup IVa (FAM labelled), and the second one for genotype IVb (HEX labelled). The specificity of the binary multiplex system was successfully evaluated against a panel of 79 VHSV strains of all genogroups and sublineages [14].

### 2.4. The Array System

For the construction of the array system, 96-well PCR strip tube plates (FrameStar Break-A-Way (4titude^®^ Limited, Surrey, UK) were employed. Four replicas of the bmRT-qPCR system (2 tubes per replica: MSI and MSII) were placed per strip, loading the corresponding mixture of primers/probes (MSI or MSII; see reference [14]) in the bottom of the tubes. The loaded volume was calculated to obtain the final concentration in the reaction as indicated below. Then, the plates were subjected to a procedure—patent pending—to fix the primers and probes to the bottom of the well. The dried plates were then stored at −25 °C until use or used immediately. After the corresponding conservation time (see below), the plates were removed from the freezer, allowed to warm to room temperature, and used immediately. 

The array was evaluated to be used for qPCR using pre-synthetized cDNA, or for RT-qPCR, using extracted viral RNA. At the time of use, the corresponding master mix was loaded, followed by the 10-fold serial dilutions (in triplicate) of each cDNA or viral RNA (respectively). Thereafter, the appropriate amplification procedure was applied, as indicated below. 

### 2.5. RNA Extraction and Quantification

Total RNA was extracted from 200 µL of clarified virus using the RNeasy mini kit (Qiagen, Madrid, Spain) following the manufacturer’s instructions, to a final elution volume of 50 µL. RNA quantification was performed with a ND-1000 spectrophotometer (Nanodrop Technologies, Wilmington, NC, USA), and its quality evaluated from the ratios A260/280 and A260/A230. Finally, the RNA concentration was adjusted to 1 ng/µL, and serial 10-fold dilutions were prepared.

### 2.6. Reverse Transcription

The cDNA synthesis was performed using Superscript III RT (GIBCO, ThermoFisher, Madrid, Spain). For this purpose, 9 µL of the extracted RNA were mixed with 2.5 ng/µL of random primers, and the mixture was incubated at 95 °C for 5 min, and then kept on ice for at least 1 min. A reverse transcription mixture containing 10 U/µL of enzyme, 0.5 mM of dNTPs, and 0.05 M dithiothreitol (DTT) in 1× ‘first strand buffer’ was then added, and the final mixture was incubated at 25 °C for 10 min, followed by 50 min at 50 °C. The reaction was finally stopped by heating at 85 °C for 5 min.

### 2.7. TaqMan Real-Time PCR

The real-time PCR was performed using the KAPA PROBE FAST universal qPCR Kit (Kapa Biosystems, SIGMA, Barcelona, Spain). The PCR mixture (25 µL final volume) contained 2.5 µL of cDNA, 12.5 µL of Kapa probe Fast Universal 2× qPCR Master Mix, and the corresponding mixture of primers/probes sets (see reference [14]), previously loaded and fixed to the PCR wells as indicated below, to obtain final concentrations of 500 nM per primer and 200 mM per probe. Following an initial 2 min activation/denaturation step at 95 °C, the mixture was subjected to 45 cycles of amplification (denaturation for 15 s at 95 °C, annealing and extension for 30 s at 58 °C), in an iCycler iQ CFX96TM Real Time System (BioRad, Madrid, Spain).

### 2.8. One Step Real-Time RT-PCR

Real-time RT-PCR was performed using QuantiFast Probe RT-PCR Master Mix without ROX (Qiagen). The reaction, with a final volume of 25 µL, contained 5 µL of RNA, 12.5 μL of 2× Master Mix, 0.25 μL of RT Mix, and the corresponding mixture of primers/probes as indicated above. All reactions were carried out in an iCycler iQ CFX96TM Real Time System (BioRad) with the following thermal profile: An initial reverse transcription step at 50 °C for 10 min, followed by a PCR activation step at 10 °C for 5 min, and 45 cycles of denaturation at 95 °C for 10 s, and annealing and extension of 30 s at an optimized temperature of 58 °C with end point fluorescence data collection.

### 2.9. Evaluation of the Efficiency and Reliability of the Array

The evaluation has been performed following stages 1 (analytical characteristic) and 3 (repeatability and reproducibility) of the OIE “Principles and methods of validation of diagnostic assays for infectious diseases” [10], testing several parameters:

Sensitivity—The sensitivity of the macroarray was evaluated in terms of the detection limit (DL), as the minimum viral titer (in TCID_50_/_mL_) detectable (by RT-qPCR and by qPCR) in the 3 replicas performed. In addition, another condition to admit the value was that the detection was performed with an average Ct below 41, and that the standard curve at that point was reliable (with R^2^ ≤ 0.95).

Specificity—The PCR procedure that the array is based on—the bmRT-qPCR—was previously validated [14] against a panel of 79 VHSV strains from all genogroups and sublineages (intragroup specificity, to ensure that any VHSV strain in detected): 2 from genogroup I, 19 from sublineage Ia, 13 from Ib, 3 from Ic, 3 from Id, and 1 from Ie; 4 from genogroup II; 12 from genogroup III; 17 from genogroup IV, sublineage a, and 5 from sublineage IVb. In addition, in that study, intergroup specificity (to ensure that only VHSV strains are detected) was evaluated against 13 non-VSHV rhabdovirus strains and an aquabinavirus. Therefore, to confirm its intra-group specificity in this macroarray, a set of 5 strains, one from each genogroup including both genogroup IV sublineages, was selected (see Section 2.1)

Repeatability and reproducibility—Three replicas per assay (all strains and dilutions) were performed with both types of assays (RT-qPCR and qPCR), to evaluate repeatability, which is defined as the precision of the procedure when replicas are simultaneously performed (the same time) on a single sample, by a certain operator, with the same method, materials, and equipment. Furthermore, taking advantage of the array stability testing, reproducibility was also tested to evaluate the precision of the array by varying the parameter ‘time’ (storage time).

Stability—As indicated above, after fixing the primer/probe sets, the arrays were used immediately (0h storage time or stored at −25 °C for 1 day, 1 week, or 3 months before being tested.

Reliability of the curves—In addition to the repeatability and reproducibility as a parameter to ensure the reliability of the arrays, an important parameter considered to evaluate the reliability of the curves was the amplification efficiency, calculated from the formula E = 10^−1/S−1^ (where S is the slope of the regression line). Although the optimum E value is 100%, values between 95 and 110% are also considered acceptable. On the other hand, to ensure the reliability of the standard curves for quantification, the coefficient of determination (R^2^) of the curves was also calculated (R^2^ ≥ 0.95 is considered acceptable, and optimum for R^2^ ≥ 0.99). For comparative purposes, in parallel to the array tested at 0 h, the normal RT-qPCR and the qPCR procedures were also carried out. 

### 2.10. Statistical Analysis

To evaluate repeatability and reproducibility (R&R), the coefficient of variation (CV = standard deviation/average × 100) from the 3 replicas and 4 repeats, respectively, was calculated. Values of CV ≤ 5% were considered indicative of high R&R, although values ≤10% were also acceptable. Besides the analysis of reproducibility to assess the effect of the storage time, a comparison of the amplification curves was carried out. To determine if two or more curves were similar, the slopes and intersections were compared using an F test, with the InStat Prism 5 statistical package (GraphPad Software Inc., La Jolla, CA, USA). If the *p*-Value is small (*p* < 0.05) for both parameters, the idea that differences are due to random sampling can be rejected and therefore both curves are different; if differences are only demonstrated for the intersections, the curves are assumed to be parallel. In addition, to confirm that the preparation of the macroarray did not affect the reliability of the standard curves, the regression lines obtained with the array tested at 0 h storage time were compared with those obtained with the normal RT-qPCR or qPCR procedures, by means of a t-test using Mann–Whitney with SGPP5; as before, significant differences were defined by *p*-Values ≤ 0.05.

## 3. Results

The macroarray designed for the detection, identification, and typing of VHSV was tested with one strain from each genogroup to be used for qPCR or for a single-step RT-qPCR, and the results are summarized in Table 1.

### 3.1. Evaluation of the Macroarray Used with RT-qPCR

As shown in the table, using the macroarray for RT-qPCR detection, the maximum viral dilution allowing positive detection was 10^−6^ with strains of any of the genogroups. Although positive amplification was obtained at dilution 10^−7^ in some cases (see Supplementary Table S1, which shows results with all dilutions and replicas), to consider the DL, a Ct value ≤ 41 in the three replicas was essential, and this was attained only with dilution 10^−6^. Considering that the stocks of virus were titrated at around 5 × 10^6^ and 5 × 10^7^ TCID_50_/_mL_ (European genogroups and genogroup IV, respectively) in EPC cells, the limit of detection was established at around 5 for the European types (I to III) and 50 for genogroup IV strains. The average Ct values from the three replicas ranged from 22, at the highest viral concentrations (Table S1), to around 39 (in most cases) at dilution 10^−6^ (Table 1). Only in one case (testing genogroup IV strains with the macroarray after three months storage), was a Ct = 40.11 reached.

**Figure 1:**
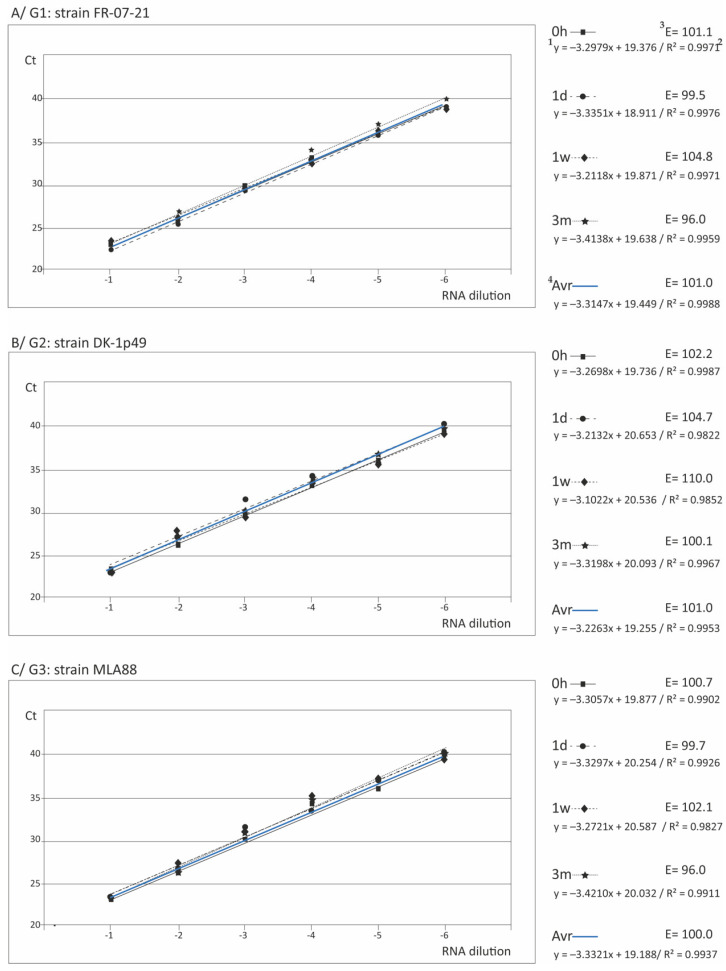

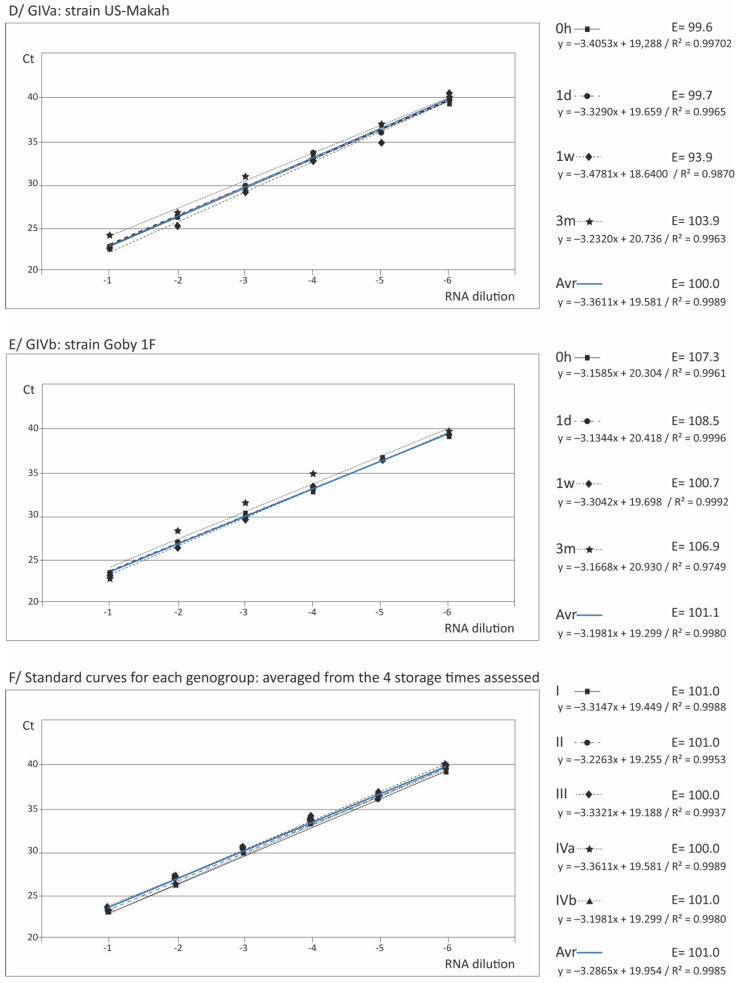
Standard curves with RT-qPCR. For each genogroup, the standard curves obtained with the macroarray applying RT-qPCR immediately after its preparation (0 h) and after the three storage times assessed (one day, one week, and three months) are shown. The equation of each curve (^1^), its reliability in terms of coefficient of determination (R^2^) (^2^), and the efficiency of the amplification (E) (^3^), calculated from the formula E = 10^−1/S^−1 (where S is the slope of the regression line), are indicated. For each genogroup, the average line (averaged from the 4 storage times) is also shown (^4^).

The reliability of the standard curves and, therefore, of the quantification, was demonstrated by R^2^ values over 0.95 in all cases (and over 0.99 in most) with all genotypes and after any storage time (Table 1), as well as with all replicas (Table S1). The standard curves and their corresponding equations are shown in Figure 1 (and Supplementary Figure S1), where the PCR reaction efficiencies (calculated from the formula E = [(10^−1/slope^−1) × 100] are also included. As observed, the E values were always within the range [96-110] with the European strains, and between 93.9 and 108.5 with strains from genotype IV, averaging 101.3 ± 0.79 and 103.45 ± 5.44, respectively, at 0 h storage, and 97.47 ± 2.29 and 105.40 ± 2.12 after 3 m at −25 °C (Table 1). Furthermore, no significant differences (*p* > 0.05) with the standard curves obtained by the regular RT-qPCR procedure were observed (data not shown).

The repeatability of the assay was evaluated using the coefficient of variation (CV) among the replicas, which showed values in most cases below 5% (except in 2 cases, which were between 5 and 10%) (Table S1). Regarding the reproducibility (calculated from the Ct data of the 4 storage times), the values were always below 5 (Table S1). To confirm reproducibility (i.e., the stability of the array at different storage times), the regression curves corresponding to the different storage times were compared to assess if significant differences between slopes and intercepts existed. As observed in Figure 1, apparently no differences were obtained between the curves corresponding to the different conservation times. This was statistically confirmed by the slopes, as shown in Table 2; only in some cases (labelled *), significant differences were observed in the intersection, mostly associated with the array conserved during three months at −25 °C. However, when the curves were analyzed, those significant differences did not seem to be so significant. In fact, studying the Ct data in Table S1 (and averages in Table 1), the differences were in most cases below 1.5, and always below 2.2. For instance, comparing the standard curves for genogroup I between 1 d and 1 w conserved arrays (Intersects: *p* = 0.0027), the Ct values differed in less than 0.92, or between 0.09 and 1.5 comparing genogroup IVa in 1 w and 3 m stored arrays (Intersect: *p* < 0.0001). Moreover, considering that the average number of Ct values associated to a change in 1 Log_10_ of dilution was 2.8 (data calculated from Table S1, but not shown), those maximum differences of 2.2 Ct would correspond to differences in viral titer always below 1 log_10_, a difference that has been considered not significant [31,32].

### 3.2. Evaluation of the Macroarray Used with qPCR

The results obtained applying qPCR are summarized in Table 1, and all the data are shown in Table S2. The Ct values ranged from 17.25 to 39.71 at the lowest and highest dilutions, respectively. Regarding the sensitivity of the procedure, with the genogroup IV strains, the DL was the same than with RT-qPCR (50 TCID_50_/_mL_); however, with the European strains the limit of detection was reduced to around 0.5 TCID_50_/_mL_. The Ct values at the DL ranges from 38.48 ± 1.11 (averaging the five strains ± standard deviation) to 38.81 ± 0.84 (depending on the storage time) with the European strains, and from 36.42 ± 1.10 to 37.68 ± 0.97 with genogroup IV strains. Regarding the reliability of the standard curves, as expected it was improved with respect to RT-qPCR, since the R^2^ values were always higher than 0.99, regardless of the viral strain, replica, and storage time (Table 1 and Table S2). Similarly, the amplification efficiency was also improved, obtaining average E values between 95 and 102. On the other hand, no significant differences (*p* > 0.05) with the standard curves obtained by the regular qPCR procedure were observed (data not shown).

**Figure 2:**
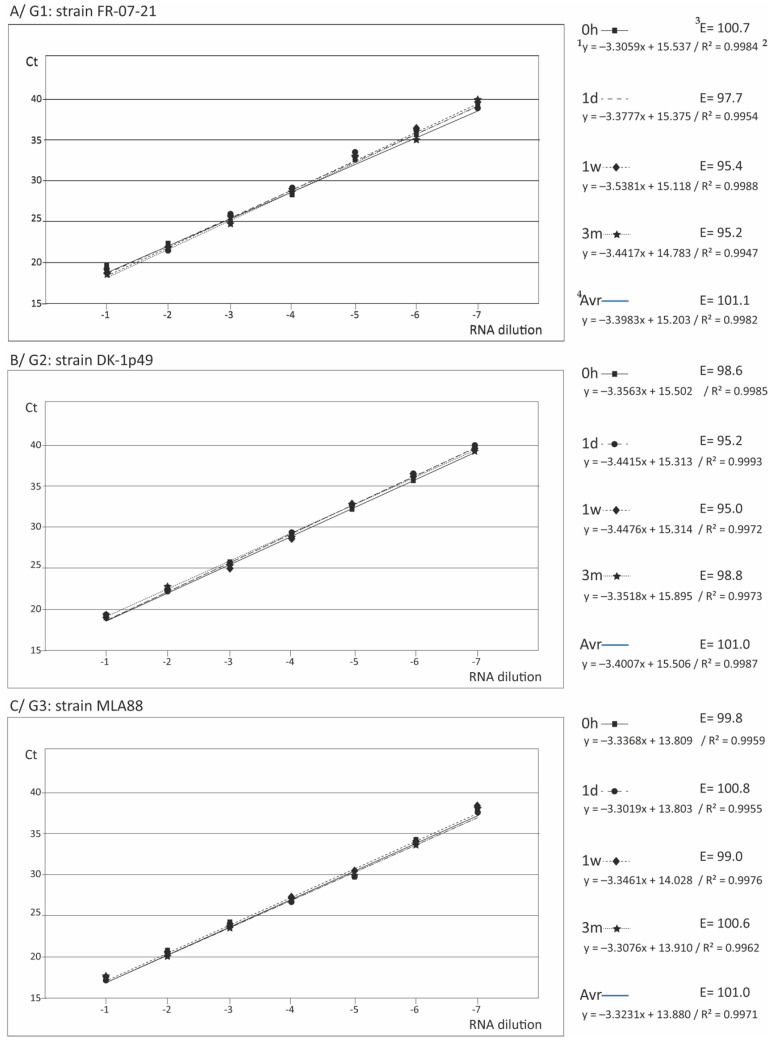

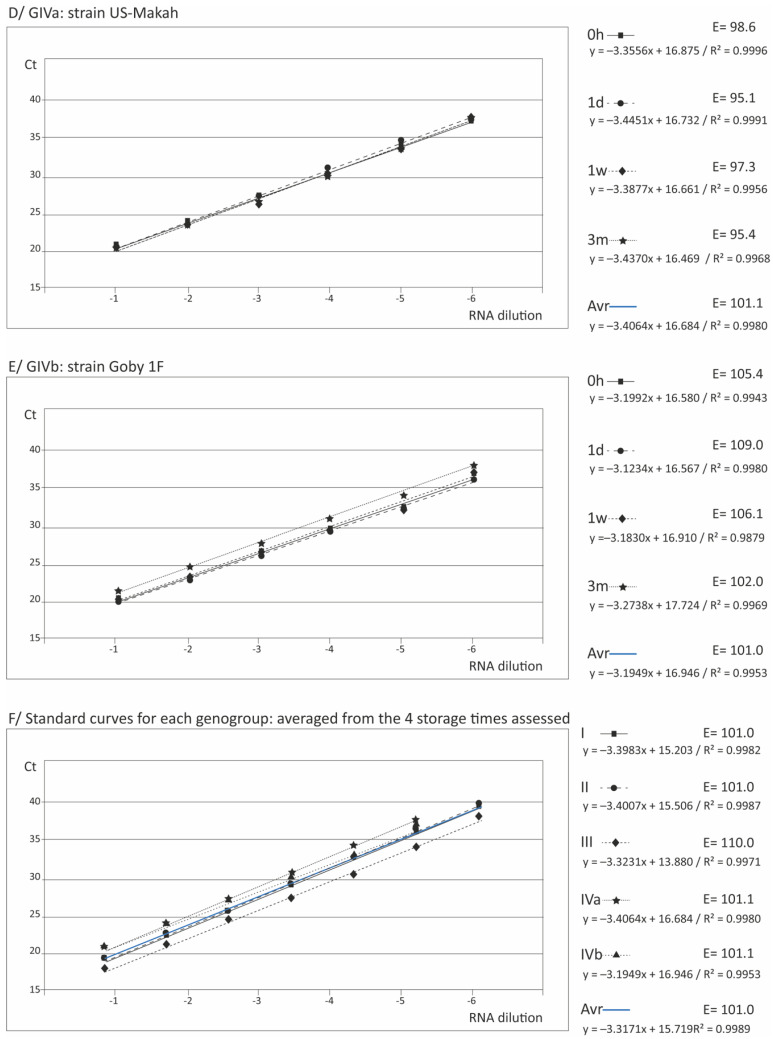
Standard curves with qPCR. For each genogroup, the standard curves obtained with the macroarray applying qPCR immediately after its preparation (0 h) and after the three storage times assessed (one day, one week, and three months) are shown. The equation of each curve (^1^), its reliability in terms of coefficient of determination (R^2^) (^2^), and the efficiency of the amplification (E) (^3^), calculated from the formula E = 10^−1/S^−1 (where S is the slope of the regression line) are indicated. For each genogroup, the average line (averaged from the 4 storage times) is also shown (^4^).

With respect to repeatability and reproducibility, the intra run CV values were below 5% in most cases (as with RT-qPCR, two values were between 5 and 10%), and the inter-run values were always below 5% (Table S2). It should be noted that, although these trials have been carried out by a researcher with 20 years of experience, in charge of the qPCR laboratory, additional trials were occasionally performed by a technician with shorter experience, yielding similar reproducibility results (results not shown). Regarding the stability of the array, as shown in Figure 2 (and Supplementary Figure S2), apparently no differences were obtained between the curves (for any viral type) corresponding to the different storage times, except for genotype IVb (Figure 2E).

The statistical analysis confirmed that there were no differences in the slopes and, only between the intercepts, certain significant differences were demonstrated in few cases, mainly with the array maintained for three months at −25 °C (Table 2).

However, the Ct data (Supplementary Table S2) revealed that, within the observed dynamic range, those differences were lower than 1 Ct in most cases, except with the genogroup IVb strain, in which case the differences in Ct reached 2.23 with respect to a 3 m storage time. Nevertheless, such differences represented less than 1 Log_10_ of titer (0.3 Log_10_ in most cases).

### 3.3. Reliability of the Standard Curves for Quantification

Having demonstrated the stability of the macroarray within the three months storage time at −25 °C, the average curves for each type strain were calculated (Figure 1F and Figure 2F) and statistically compared. As shown in Table 3, no significant differences among the slopes were observed in most cases, both with qPCR and RT-qPCR, which demonstrates that the curves are parallel. The exception was the genotype IVb strain, whose standard curve slope showed significant differences with the rest, except the genotype III strain. However, significant differences were observed, in most cases, between the intersections of the curves.

## 4. Discussion

The use of real-time PCR in viral diagnosis is as common nowadays as normal PCR was in the late 1990s. Its high sensitivity, specificity, and robustness is well known, but it also reduces the diagnostic time and minimizes the risk of carryover contamination. However, the handicap of this technology comes about when diagnosis and typing of multiple samples in necessary: Unless an expensive robotic equipment is available, the process of preparing the PCR mixes corresponding to each sample is tedious and there is a risk of cross contamination. A second issue comes up when multiple pathogens must be detected in a single sample; this can be partially solved with a multiplex strategy, which needs extensive research to ensure that interference between the different markers does not reduce the reliability of the procedure. However, such strategy is quite limited by the number of markers that can be used in a single reaction.

In the last decade, a new technology has become quite popular [33,34], namely DNA arrays or chips, which allows the detection of a large number of pathogens in a single assay; this solves the second issue, but not the first. Moreover, that technology is based on nucleic acid hybridization (NAH), which reduces the sensitivity of the diagnosis [18] and scientists therefore frequently combine it with previous amplification by PCR [22,23,35,36].

Other approaches combine both methodologies into one: qPCR-based macroarrays. The use of this kind of array for viral detection has been reported by very few authors. Venter et al. [21] designed a macroarray for diagnosis of meningoencephalitis, mostly of viral etiology; however, that procedure actually requires an initial application of PCR to the extracted nucleic acid, and then the array is used just for the detection by NAH. The real concept of a PCR-based macroarray has been applied by Hasan et al. [20] for respiratory pathogens, and by Ries et al. [19] for bluetongue virus. In both cases, 96-well PCR plates are filled with the corresponding mixture of primers and probes, in a solution, and the plates can be stored at −20 °C until use; however, prior to their use, the plates must be thawed and briefly centrifuged.

The method we have designed and validated here is similar to those described above, although the first difference comes from the fact that the primer/probe mixture is not in a liquid format in the well, but rather dried fixed to the bottom; therefore, thawing is quick, and the plate does not need to be centrifuged to pellet the mixture. In addition, although Ries et al. [19] ensured that the plates can be stored for months without affecting sensitivity, they did not actually provide any evidence. In the present report, we have demonstrated that the macroarray plate can be stored for one week obtaining the same Ct values, and for three months with no loss of sensitivity: The Ct values at the highest dilution increased between 1 and 2 using the array with RT-qPCR, and even less with qPCR. Furthermore, we tested the array in a single assay after one year of storage and observed a reduction of sensitivity of just 1 Log_10_ with strains from the genogroup IV, but not with the European types (results not shown). The importance of this feature of our macroarray for the organizational capacity of a diagnostic laboratory is obvious.

Regarding the sensitivity of the macroarray, initially we expected to obtain the same DL with it than with the PCR procedure that was based on the bmRT-qPCR reported by Vázquez et al. [14]. In fact, in that study, the minimum detectable viral titer was between 3.1 and 39 TCID_50_/_mL_ with the European genogroups, and around 56 with the strains from genogroup IV. The results obtained here with the macroarray were quite similar to the original bmRT-qPCR system [14], and even improved with qPCR with the European strains. The validation of other arrays has resulted in higher DL values. For instance, Tian et al. [35] designed a microarray for detection and discrimination of A and B types of flu virus, and reported a DL of 10^2^ copies/µL; but that value actually corresponds to 10^5^ copies/mL, markedly higher than the DL of our macroarrray. Lien et al. [24], using a chip for fish viruses (and bacteria), obtained a detection limit of 10 genome copies per microliter (10^4^ copies/mL), similar to what was obtained by Lievens et al. [22] with an array for three cyprinid herpesviruses. With respect to arrays designed for human viruses, high DL values have also been reported, from the 1 × 10^2^ cps/µL (10^5^ cps/mL) with a microarray for flu detection [35], to an average of 6.36 cps/reaction (1.3 × 10^2^ cps/mL) with a macroarray designed for a variety of human viruses [21]. 

The macroarray would be useless if it were demonstrated to be inappropriate for some viral types. However, its sensitivity did not change within the European strains, and only differed 1 Log_10_ with respect to the American and Asian types. Additionally, as already indicated, the real-time system employed for the array –the bmRT-qPCR system– had previously been successfully tested against a panel of 79 VHSV strains from all genogroups and sublineages. Moreover, the binary system combined with the use of specific probes for each genotype has the advantage of the typing the VHSV strain simultaneously with its detection. Besides, its high repeatability, demonstrated by the low CV values between replicas, ensures that the use of a single replica—a practice that is certainly not uncommon in diagnostic laboratories—does not jeopardize the reliability of the result. Regarding specificity, in none of those reports cited above, the intragroup specificity is ensured since different types within each group assessed was not tested in most cases. Neither was reproducibility tested in any of them. 

The reliability of the standard curves, demonstrated by the R^2^ and E values, ensures the reliability of the quantification using the corresponding standard for each genotype. Nevertheless, since the slope of the curve for the genogroup III strain was demonstrated not to differ from the remaining types, it could be used as a standard curve for quantification of VHSV strains from any type, of course within the dynamic range obtained in this study. Nevertheless, it is well known that extrapolating over or below the dynamic range is risky in any case, since it compromises the reliability of the quantification. Finally, this array has been validated against crude virus of reference strains, and under an analytical point of view. A large diagnostic evaluation is at present been designed as a ring test, to assess the reliability of the macroarray directly on infected fish tissue and will be part of a further report.

## 5. Conclusions

Based on these results, we are convinced that this macroarray will be extremely useful in all types of fish viral pathology diagnostic laboratories, in this specific case for the detection, typing, and quantification of VHSV, and it will shortly be validated for other viruses of interest in this field.

## Figures and Tables

**Table 1:** Reliability of the macroarray: Summary of results.

Strg ^1^			a/ RT-qPCR						b/ qPCR		
			^7^ G I. II & III		G. IV				G I. II & III		G. IV
0 h	^2^ Dil		10^−6^		10^−6^		Dil		10^−7^		10^−6^
	^3^ DL		5 TCID_50_/mL		50 TCID_50_/mL		LD		0.5 TCID_50_/mL		50 TCID_50_/mL
	^4^ Ct		39.21 ± 0.36		39.03 ± 0.23		Ct		38.65 ± 0.86		36.72 ± 0.47
	^5^ E		[101.0-102.2] 101.30 ± 0.79		[99.6-107.3] 103.45 ± 5.44		E		[98.6-100.7] 99.70 ± 1.05		[98.6-105.4] 102.0 ± 4.81
	^6^ R^2^		0.9953 ± 0.0045		0.9966 ± 0.0006		R^2^		0.9963 ± 0.0019		0.9970 ± 0.0037
1 d	Dil		10^−6^		10^−6^		Dil		10^−7^		10^−6^
	DL		5 TCID_50_/mL		50 TCID_50_/mL		LD		0.5 TCID_50_/mL		50 TCID_50_/mL
	Ct		39.61 ± 0.86		39.38 ± 0.37		Ct		38.48 ± 1.11		36.42 ± 1.10
	E		[99.5-104.7] 101.30 ± 2.98		[99.7-108.5] 104.10 ± 6.22		E		[99.2-100.8] 97.90 ± 2.81		[95.1-109.0] 102.05 ± 9.83
	R^2^		0.9908 ± 0.0078		0.9980 ± 0.0022		R^2^		0.9967 ± 0.0022		0.9985 ± 0.0008
1 w	Dil		10^−6^		10^−6^		Dil		10^−7^		10^−6^
	DL		5 TCID_50_/mL		50 TCID_50_/mL		LD		0.5 TCID_50_/mL		50 TCID_50_/mL
	Ct		39.16 ± 0.16		37.46 ± 3.37		Ct		38.87 ± 0.84		37.23 ± 0.32
	E		[102.1-110.0] 105.33 ± 4.52		[93.9-100.7] 97.30 ± 4.81		E		[95.0-99.0] 96.47 ± 2.20		[97.3-106.1] 101.70 ± 6.22
	R^2^		0.9883 ± 0.0077		0.9931 ± 0.0086		R^2^		0.9979 ± 0.0009		0.9997 ± 0.0055
3 m	Dil		10^−6^		10^−6^		Dil		10^−7^		10^−6^
	DL		5 TCID_50_/mL		50 TCID_50_/mL		LD		0.5 TCID_50_/mL		50 TCID_50_/mL
	Ct		39.75 ± 0.03		40.11 ± 0.03		Ct		38.85 ± 0.97		37.68 ± 0.97
	E		[96.0-100.1] 97.47 ± 2.29		[103.9-106.9] 105.40 ± 2.12		E		[95.2-100.6] 98.20 ± 2.75		[95.4-102.0] 98.7 ± 4.67
	R^2^		0.9996 ± 0.0030		0.9856 ± 0.1513		R^2^		0.9961 ± 0.0013		0.9969 ± 0.0001

^1^ Strg: Storage time; ^2^ Dil: Dilution; ^3^ DL: Detection limit; ^4^ Ct: Average Ct values ± standard deviation; ^5^ E: Efficiency of the amplification, calculated from the formula E = 10^−1/S^−1 (where S is the slope of the regression line), shown as range (between square brackets) and average ± standard deviation; ^6^ R^2^: coefficient of determination (R^2^) of the curves; ^7^ G: Genogroup.

**Table 2:** Statistical analysis of the effect of storage times on the stability of the array.

		RT-qPCR								qPCR						
		GI								GI						
		0 h		1 d		1 w		3 m		0 h		1 d		1 w		3 m
^3^ GI	0 h	**-**		0.6870 ^1^ 0.0572 ^2^		0.4279 0.2960		0.2330 0.0003		**-**		0.3392 0.4122		0.0880 0.4912		0.0913 0.6363
	1 d			**-**		0.1999 0.0027 *		0.3355 <0.0001 *				**-**		0.4671 0.9243		0.3581 0.2913
	1 w					**-**		0.0473 *						**-**		0.7441 0.3262
	3 m							**-**								**-**
		**GII**								**GII**						
		0 h		1 d		1 w		3 m		0 h		1 d		1 w		3 m
GII	0 h	**-**		0.7007 0.0062 *		0.2154 0.2935		0.6276 0.0041 *		**-**		0.1941 0.2514		0.1730 0.1646		0.9426 0.0054 *
	1 d			**-**		0.4612 0.1647		0.4614 0.4454				**-**		0.8651 0.7263		0.1669 0.0917
	1 w					**-**		0.1144 0.3423						**-**		0.1499 0.2193
	3 m							**-**								**-**
		**GIII**								**GIII**						
		0 h		1 d		1 w		3 m		0 h		1 d		1 w		3 m
GIII	0 h	**-**		0.8874 0.1127		0.8706 0.0942		0.4591 0.0396 *		**-**		0.7884 0.5733		0.9183 0.1650		0.7878 0.9543
	1 d			**-**		0.7924 0.7207		0.5924 0.7339				**-**		0.7364 0.1266		0.9692 0.6456
	1 w					**-**		0.4752 0.9250						**-**		0.7264 0.2148
	3 m							**-**								**-**
		**GIVa**								**GIVa**						
		0 h		1 d		1 w		3 m		0 h		1 d		1 w		3 m
GIVa	0 h	**-**		0.5133 0.5999		0.6104 0.1040		0.1462 0.0002 *		**-**		0.2099 0.1683		0.6969 0.4638		0.3377 0.4003
	1 d			**-**		0.3190 0.0569		0.4383 0.0013 *				**-**		0.4641 0.0467 *		0.9200 0.0369 *
	1 w					**-**		0.1061 <0.0001 *						**-**		0.5878 0.8919
	3 m							**-**								**-**
		**GIVb**								**GIVb**						
		0 h		1 d		1 w		3 m		0 h		1 d		1 w		3 m
GIVb	0 h	**-**		0.8433 0.8850		0.1548 0.5818		0.9618 0.0262 *		**-**		0.4392 0.0992		0.9016 0.2244		0.4585 <0.0001 *
	1 d			**-**		0.1433 0.5257		0.8549 0.0418 *				**-**		0.6623 0.0210*		0.1596 <0.0001 *
	1 w					**-**		0.4018 0.0100 *						**-**		0.5123 <0.0001 *
	3 m							**-**								**-**

The slopes (^1^) and intersections (^2^) were compared by an F test, using the InStat Prism statistical package (Prism 5, GraphPad Software Inc., La Jolla, CA, USA). The *p*-Values are shown; if *p* < 0.05 (*) for both parameters, the idea that differences are due to random sampling can be rejected and therefore both curves are different; if differences are only demonstrated for the intersections, the curves are assumed to be parallel. ^3^ Genogroups.

**Table 3:** Comparison of the quantification curves among genogroups.

		RT-qPCR										qPCR								
^3^ Genogr.		GI		GII		GIII		GIVa		GIVb		GI		GII		GIII		GIVa		GIVb
GI		-		0.2598 ^1^		0.7936		0.4375		0.0689		-		0.9678		0.2650		0.8943		0.0109
		-		0.0006 ^2,^*		<0.0001 *		0.0174 *		0.0003 *		-		0.0072 *		<0.0001		<0.0001		**
GII		-		-		0.2880		0.1132		0.7380		-		-		0.1951		0.9039		0.0036
		-		-		0.0960		0.1593		0.6605		-		-		<0.0001		<0.0001		**
GIII		-		-		-		0.7584		0.1409		-		-		-		0.2150		0.1161
		-		-		-		0.0029 *		0.0300 *		-		-		-		<0.0001		<0.0001
GIVa		-		-		-		-		0.0280 *		-		-		-		-		0.0059
		-		-		-		-		**		-		-		-		-		**
GIVb		-		-		-		-		-		-		-		-		-		-
		-		-		-		-		-		-		-		-		-		-

The slopes (^1^) and intersection (^2^) were compared by an F test, using the InStat Prism statistical package (Prism 5, GraphPad Software Inc., La Jolla, CA, USA). The *p*-Values are shown; if *p* < 0.05 (*) for both parameters, the idea that differences are due to random sampling can be rejected and therefore both curves are different; if differences are only demonstrated for the intersections, the curves are assumed to be parallel; if the slopes differ significantly, it is not possible to test whether the intercepts differ (**). ^3^ Genogroups.

## Data Availability

The data presented in this study are available in Supplementary Material here.

## Supplementary Materials

The following are available online at https://www.mdpi.com/2076-2615/11/3/841/s1, Table S1: Data of RT-qPCR and regression lines for all replicas and repeats; Table S2: Data of qPCR and regression lines for all replicas and repeats; Figure S1: Standard curves with RT-qPCR, showing the 3 replicas; Figure S2: Standard curves with qPCR, showing the 3 replicas.
