# Supplementary material for: Design and Evaluation of a Macroarray for Detection, Identification, and Typing of Viral Hemorrhagic Septicemia Virus (VHSV)
Source: Animals (Basel). 2021 Mar 16;11(3):841. doi: 10.3390/ani11030841 (PMC8002285; doi:10.3390/ani11030841)
Supplement: Supplementary file 1 [file animals-11-00841-s001.zip › Sppl Files/Sppl Table DOCs/Supplementary Table S2.docx]

Supplementary Table S2.- Data of qPCR and regression lines for all replicas and repeats

|  |  | ^1^G1: FR-07-21 | | | | | | | | | | | | | | | | | | | | | | | | | | | | | | | | | | | | | |  | |  | |  | |  | | |  |
| --- | --- | --- | --- | --- | --- | --- | --- | --- | --- | --- | --- | --- | --- | --- | --- | --- | --- | --- | --- | --- | --- | --- | --- | --- | --- | --- | --- | --- | --- | --- | --- | --- | --- | --- | --- | --- | --- | --- | --- | --- | --- | --- | --- | --- | --- | --- | --- | --- | --- |
| ^2^Dil. |  | ^3^0 h | | | | | | | | |  | 1 d | | | | | | | | |  | 1 w | | | | | | | | |  | 3 m | | | | | | | |  | | Reproducibility^9^ | | | | | | |  |
|  |  | ^4^Rpl 1 | Rpl 2 | Rpl 3 | ^5^Avrg. | | ^6^Desv | ^7^CV |  | Rpl 1 | | | Rpl 2 | Rpl 3 | Avrg. | | Desv | CV |  | Rpl 1 | | | Rpl 2 | Rpl 3 | Avrg. | | Desv | CV |  | Rpl 1 | | | Rpl 2 | Rpl 3 | Avrg. | | Desv | CV |  | | Avrg. | | Desv | | CV | |  |  |  |
| -1 |  | 19.29 | 18.97 | 19.31 | 19.19 | | 0.19 | 0.99 |  | 19.27 | | | 19.26 | 18.76 | 19.10 | | 0.29 | 1.53 |  | 19.34 | | | 18.37 | 18.55 | 18.75 | | 0.52 | 2.75 |  | 19.19 | | | 18.52 | 18.23 | 18.65 | | 0.49 | 2.64 |  | | 18.92 | | 0.41 | | 2.18 | |  |  |  |
| -2 |  | 22.32 | 21.50 | 22.15 | 21.99 | | 0.43 | 1.97 |  | 21.69 | | | 21.32 | 21.96 | 21.66 | | 0.32 | 1.48 |  | 22.79 | | | 21.20 | 21.36 | 21.78 | | 0.88 | 4.02 |  | 21.26 | | | 22.61 | 21.23 | 21.70 | | 0.79 | 3.63 |  | | 21.78 | | 0.57 | | 2.61 | |  |  |  |
| -3 |  | 25.16 | 25.24 | 25.22 | 25.20 | | 0.04 | 0.17 |  | 25.45 | | | 25.24 | 25.38 | 25.36 | | 0.11 | 0.42 |  | 25.63 | | | 25.03 | 25.17 | 25.28 | | 0.31 | 1.24 |  | 23.82 | | | 25.13 | 25.11 | 24.69 | | 0.75 | 3.04 |  | | 25.13 | | 0.44 | | 1.77 | |  |  |  |
| -4 |  | 28.12 | 28.57 | 28.59 | 28.43 | | 0.27 | 0.95 |  | 28.83 | | | 28.52 | 28.29 | 28.55 | | 0.27 | 0.95 |  | 28.95 | | | 28.73 | 28.67 | 28.78 | | 0.15 | 0.51 |  | 28.03 | | | 28.47 | 28.67 | 28.39 | | 0.33 | 1.15 |  | | 28.54 | | 0.28 | | 0.96 | |  |  |  |
| -5 |  | 32.80 | 32.22 | 32.42 | 32.48 | | 0.30 | 0.91 |  | 32.98 | | | 33.13 | 33.21 | 33.11 | | 0.12 | 0.35 |  | 32.66 | | | 32.08 | 32.76 | 32.50 | | 0.37 | 1.13 |  | 32.57 | | | 33.01 | 32.99 | 32.86 | | 0.25 | 0.76 |  | | 32.74 | | 0.36 | | 1.10 | |  |  |  |
| -6 |  | 35.09 | 34.94 | 35.87 | 35.30 | | 0.50 | 1.41 |  | 35.69 | | | 35.92 | 36.03 | 35.88 | | 0.17 | 0.48 |  | 35.83 | | | 36.32 | 36.21 | 36.12 | | 0.26 | 0.71 |  | 34.65 | | | 34.90 | 35.01 | 34.85 | | 0.18 | 0.53 |  | | 35.54 | | 0.58 | | 1.63 | |  |  |  |
| -7 |  | 38.26 | 38.56 | 39.43 | 38.75 | | 0.61 | 1.57 |  | 38.24 | | | 38.56 | 38.87 | 38.56 | | 0.32 | 0.82 |  | 38.03 | | | 38.75 | 39.85 | 38.88 | | 0.92 | 2.36 |  | 38.57 | | | 39.37 | 40.73 | 39.56 | | 1.09 | 2.76 |  | | 38.93 | | 0.78 | | 2.01 | |  |  |  |
| -8 |  | - | - | - | - | | - | - |  | - | | | - | 41.09 | - | | - | - |  | - | | | 40.81 | - | - | | - | - |  | - | | | - | - | - | | - | - |  | | - | | - | | - | |  |  |  |
|  |  |  |  |  |  | |  |  |  |  | | |  |  |  | |  |  |  |  | | |  |  |  | |  |  |  |  | | |  |  |  | |  |  |  | |  | |  | |  | |  |  |  |
| Rpl 1 |  | y = 3.2171x + 15.849^8^ | | | | R² = 0.99554 | | | | |  | y = 3.3014x + 15.673 | | | | R² = 0.99535 | | | | |  | y = 3.1850x + 16.293 | | | | R² = 0.99727 | | | | |  | y = 3.3454x + 14.917 | | | | R² = 0.98937 | | | |  | |  | |  | |  | | |  |
| Rpl 2 |  | y = 3.3079x + 15.338 | | | | R² = 0.99839 | | | | |  | y = 3.3925x + 15.280 | | | | R² = 0.99339 | | | | |  | y = 3.5154x + 14.579 | | | | R² = 0.99747 | | | | |  | y = 3.3932x + 15.286 | | | | R² = 0.99439 | | | |  | |  | |  | |  | | |  |
| Rpl 3 |  | y = 3.3927x + 15.425 | | | | R² = 0.99806 | | | | |  | y = 3.4393x + 15.171 | | | | R² = 0.99594 | | | | |  | y = 3.6139x + 14.483 | | | | R² = 0.99870 | | | | |  | y = 3.6764x + 14.147 | | | | R² = 0.99308 | | | |  | |  | |  | |  | | |  |
|  |  |  |  |  |  | |  |  |  |  | | |  |  |  | |  |  |  |  | | |  |  |  | |  |  |  |  | | |  |  |  | |  |  |  | |  | |  | |  | |  |  |  |
| Avrg. |  | y = 3.3059x + 15.537 | | | | R² = 0.99836 | | | | |  | y = 3.3777x + 15.375 | | | | R² = 0.99544 | | | | |  | y = 3.4381x + 15.118 | | | | R² = 0.99883 | | | | |  | y = 3.4717x + 14.783 | | | | R² = 0.99468 | | | |  | |  | |  | |  | | |  |
|  |  | G2: DK-1p49 | | | | | | | | | | | | | | | | | | | | | | | | | | | | | | | | | | | | | |  | |  | |  | |  | | |  |
| Dil. |  | 0 h | | | | | | | | |  | 1 d | | | | | | | | |  | 1 w | | | | | | | | |  | 3 m | | | | | | | |  | | Reproducibility | | | | | | |  |
|  |  | Rpl 1 | Rpl 2 | Rpl 3 | Avrg. | | Desv | CV |  | Rpl 1 | | | Rpl 2 | Rpl 3 | Avrg. | | Desv | CV |  | Rpl 1 | | | Rpl 2 | Rpl 3 | Avrg. | | Desv | CV |  | Rpl 1 | | | Rpl 2 | Rpl 3 | Avrg. | | Desv | CV |  | | Avrg. | | Desv | | CV | |  |  |  |
| -1 |  | 19.24 | 19.05 | 19.11 | 19.13 | | 0.10 | 0.51 |  | 19.14 | | | 18.98 | 19.06 | 19.06 | | 0.08 | 0.42 |  | 18.93 | | | 19.09 | 19.23 | 19.08 | | 0.15 | 0.79 |  | 19.86 | | | 19.28 | 19.54 | 19.56 | | 0.29 | 1.49 |  | | 19.21 | | 0.26 | | 1.36 | |  |  |  |
| -2 |  | 22.01 | 22.03 | 22.77 | 22.27 | | 0.43 | 1.94 |  | 22.01 | | | 22.03 | 22.36 | 22.13 | | 0.20 | 0.89 |  | 22.35 | | | 22.47 | 22.81 | 22.54 | | 0.24 | 1.06 |  | 22.61 | | | 22.77 | 22.86 | 22.75 | | 0.13 | 0.56 |  | | 22.42 | | 0.34 | | 1.52 | |  |  |  |
| -3 |  | 25.41 | 25.50 | 25.33 | 25.41 | | 0.09 | 0.33 |  | 25.80 | | | 25.60 | 24.73 | 25.38 | | 0.57 | 2.24 |  | 25.29 | | | 25.25 | 24.96 | 25.17 | | 0.18 | 0.72 |  | 25.51 | | | 25.39 | 25.40 | 25.43 | | 0.07 | 0.26 |  | | 25.35 | | 0.28 | | 1.11 | |  |  |  |
| -4 |  | 28.68 | 29.12 | 28.46 | 28.75 | | 0.34 | 1.17 |  | 28.86 | | | 29.09 | 28.95 | 28.97 | | 0.12 | 0.40 |  | 28.90 | | | 28.19 | 28.39 | 28.49 | | 0.37 | 1.28 |  | 29.01 | | | 28.69 | 28.77 | 28.82 | | 0.17 | 0.58 |  | | 28.76 | | 0.29 | | 1.01 | |  |  |  |
| -5 |  | 31.86 | 32.09 | 31.95 | 31.97 | | 0.12 | 0.36 |  | 32.06 | | | 32.28 | 32.84 | 32.39 | | 0.40 | 1.24 |  | 33.14 | | | 32.03 | 33.01 | 32.73 | | 0.61 | 1.85 |  | 33.03 | | | 32.69 | 32.97 | 32.90 | | 0.18 | 0.55 |  | | 32.50 | | 0.49 | | 1.52 | |  |  |  |
| -6 |  | 35.22 | 36.00 | 35.27 | 35.50 | | 0.44 | 1.23 |  | 36.16 | | | 35.23 | 36.84 | 36.08 | | 0.81 | 2.24 |  | 36.04 | | | 36.51 | 35.94 | 36.16 | | 0.30 | 0.84 |  | 36.23 | | | 36.69 | 36.28 | 36.40 | | 0.25 | 0.69 |  | | 36.03 | | 0.55 | | 1.53 | |  |  |  |
| -7 |  | 39.19 | 40.24 | 38.94 | 39.46 | | 0.69 | 1.75 |  | 39.87 | | | 39.45 | 39.32 | 39.55 | | 0.29 | 0.73 |  | 39.93 | | | 39.73 | 39.48 | 39.71 | | 0.23 | 0.57 |  | 39.64 | | | 38.96 | 39.16 | 39.25 | | 0.35 | 0.89 |  | | 39.49 | | 0.40 | | 1.02 | |  |  |  |
| -8 |  | - | - | 40.93 | - | | - | - |  | - | | | - | - | - | | - | - |  | - | | | - | - | - | | - | - |  | - | | | - | - | - | | - | - |  | | - | | - | | - | |  |  |  |
|  |  |  |  |  |  | |  |  |  |  | | |  |  |  | |  |  |  |  | | |  |  |  | |  |  |  |  | | |  |  |  | |  |  |  | |  | |  | |  | |  |  |  |
| Rpl 1 |  | y = 3.3114x + 15.556 | | | | R² = 0.99837 | | | | |  | y = 3.4554x + 15.307 | | | | R² = 0.99790 | | | | |  | y = 3.5082x + 15.193 | | | | R² = 0.99826 | | | | |  | y = 3.3607x + 15.970 | | | | R² = 0.99735 | | | |  | |  | |  | |  | | |  |
| Rpl 2 |  | y = 3.5036x + 15.133 | | | | R² = 0.99744 | | | | |  | y = 3.3746x + 15.453 | | | | R² = 0.99863 | | | | |  | y = 3.4564x + 15.213 | | | | R² = 0.99477 | | | | |  | y = 3.3636x + 15.756 | | | | R² = 0.99596 | | | |  | |  | |  | |  | | |  |
| Rpl 3 |  | y = 3.2539x + 15.817 | | | | R² = 0.99817 | | | | |  | y = 3.4946x + 15.179 | | | | R² = 0.99500 | | | | |  | y = 3.3950x + 15.537 | | | | R² = 0.99472 | | | | |  | y = 3.3311x + 15.959 | | | | R² = 0.99698 | | | |  | |  | |  | |  | | |  |
|  |  |  |  |  |  | |  |  |  |  | | |  |  |  | |  |  |  |  | | |  |  |  | |  |  |  |  | | |  |  |  | |  |  |  | |  | |  | |  | |  |  |  |
| Avrg. |  | y = 3.3563x + 15.502 | | | | R² = 0.99852 | | | | |  | y = 3.4415x + 15.313 | | | | R² = 0 99931 | | | | |  | y = 3.4532x + 15.314 | | | | R² = 0.99716 | | | | |  | y = 3.3518x + 15.895 | | | | R² = 0.99733 | | | |  | |  | |  | |  | | |  |
|  |  | G3: MLA88 | | | | | | | | | | | | | | | | | | | | | | | | | | | | | | | | | | | | | |  | |  | |  | |  | | |  |
| Dil. |  | 0 h | | | | | | | | |  | 1 d | | | | | | | | |  | 1 w | | | | | | | | |  | 3 m | | | | | | | |  | | Reproducibility | | | | | | |  |
|  |  | Rpl 1 | Rpl 2 | Rpl 3 | Avrg. | | Desv | CV |  | Rpl 1 | | | Rpl 2 | Rpl 3 | Avrg. | | Desv | CV |  | Rpl 1 | | | Rpl 2 | Rpl 3 | Avrg. | | Desv | CV |  | Rpl 1 | | | Rpl 2 | Rpl 3 | Avrg. | | Desv | CV |  | | Avrg. | | Desv | | CV | |  |  |  |
| -1 |  | 17.26 | 17.27 | 17.21 | 17.25 | | 0.03 | 0.19 |  | 17.28 | | | 17.26 | 17.38 | 17.31 | | 0.06 | 0.37 |  | 17.32 | | | 17.82 | 18.07 | 17.74 | | 0.38 | 2.15 |  | 18.01 | | | 17.59 | 17.74 | 17.78 | | 0.21 | 1.20 |  | | 17.52 | | 0.32 | | 1.80 | |  |  |  |
| -2 |  | 20.46 | 20.55 | 20.96 | 20.66 | | 0.27 | 1.29 |  | 20.88 | | | 20.45 | 20.21 | 20.51 | | 0.34 | 1.65 |  | 20.10 | | | 21.05 | 21.28 | 20.81 | | 0.63 | 3.01 |  | 20.50 | | | 20.17 | 20.29 | 20.32 | | 0.17 | 0.82 |  | | 20.58 | | 0.38 | | 1.86 | |  |  |  |
| -3 |  | 24.20 | 24.07 | 24.25 | 24.17 | | 0.09 | 0.38 |  | 24.21 | | | 24.20 | 23.58 | 24.00 | | 0.36 | 1.50 |  | 23.06 | | | 24.04 | 24.45 | 23.85 | | 0.71 | 2.99 |  | 24.11 | | | 23.46 | 23.24 | 23.60 | | 0.45 | 1.92 |  | | 23.91 | | 0.45 | | 1.88 | |  |  |  |
| -4 |  | 26.64 | 26.90 | 26.53 | 26.69 | | 0.19 | 0.71 |  | 26.68 | | | 26.39 | 26.39 | 26.49 | | 0.17 | 0.63 |  | 26.98 | | | 27.19 | 27.21 | 27.13 | | 0.13 | 0.47 |  | 27.52 | | | 26.76 | 26.88 | 27.05 | | 0.41 | 1.51 |  | | 26.84 | | 0.35 | | 1.29 | |  |  |  |
| -5 |  | 29.96 | 29.52 | 29.71 | 29.73 | | 0.22 | 0.74 |  | 29.84 | | | 29.79 | 28.82 | 29.48 | | 0.58 | 1.95 |  | 30.63 | | | 30.32 | 30.22 | 30.39 | | 0.21 | 0.70 |  | 30.21 | | | 30.14 | 29.65 | 30.00 | | 0.31 | 1.02 |  | | 29.90 | | 0.47 | | 1.56 | |  |  |  |
| -6 |  | 33.39 | 34.91 | 33.26 | 33.85 | | 0.92 | 2.71 |  | 35.77 | | | 34.42 | 31.65 | 33.95 | | 2.10 | 6.19 |  | 33.01 | | | 34.88 | 33.93 | 33.94 | | 0.94 | 2.75 |  | 33.58 | | | 33.37 | 33.50 | 33.48 | | 0.11 | 0.32 |  | | 33.81 | | 1.07 | | 3.18 | |  |  |  |
| -7 |  | 37.99 | 38.14 | 37.09 | 37.74 | | 0.57 | 1.50 |  | 39.02 | | | 37.42 | 35.58 | 37.34 | | 1.72 | 4.61 |  | 38.64 | | | 37.96 | 37.50 | 38.03 | | 0.57 | 1.51 |  | 36.11 | | | 37.24 | 39.88 | 37.74 | | 1.93 | 5.13 |  | | 37.71 | | 1.18 | | 3.14 | |  |  |  |
| -8 |  | - | 41.13 | 40.31 | - | | - | - |  | - | | | 41.05 | 40.73 | - | | - | - |  | - | | | 41.01 | 40.97 | - | | - | - |  | 40.11 | | | - | - | - | | - | - |  | | - | | - | | - | |  |  |  |
|  |  |  |  |  |  | |  |  |  |  | | |  |  |  | |  |  |  |  | | |  |  |  | |  |  |  |  | | |  |  |  | |  |  |  | |  | |  | |  | |  |  |  |
| Rpl 1 |  | y = 3.3504x + 13.727 | | | | R² = 0.99503 | | | | |  | y = 3.5939x + 13.293 | | | | R² = 0.98773 | | | | |  | y = 3.4768x + 13.199 | | | | R² = 0.99098 | | | | |  | y = 3.0914x + 14.783 | | | | R² = 0.99800 | | | |  | |  | |  | |  | | |  |
| Rpl 2 |  | y = 3.4564x + 13.511 | | | | R² = 0.99235 | | | | |  | y = 3.3575x + 13.703 | | | | R² = 0.99484 | | | | |  | y = 3.3700x + 14.129 | | | | R² = 0.99647 | | | | |  | y = 3.2868x + 13.814 | | | | R² = 0.99806 | | | |  | |  | |  | |  | | |  |
| Rpl 3 |  | y = 3.2036x + 14.187 | | | | R² = 0.99623 | | | | |  | y = 2.9543x + 14.413 | | | | R² = 0.99698 | | | | |  | y = 3.1914x + 14.757 | | | | R² = 0.99806 | | | | |  | y = 3.5446x + 13.133 | | | | R² = 0.97884 | | | |  | |  | |  | |  | | |  |
|  |  |  |  |  |  | |  |  |  |  | | |  |  |  | |  |  |  |  | | |  |  |  | |  |  |  |  | | |  |  |  | |  |  |  | |  | |  | |  | |  |  |  |
| Avrg. |  | y = 3.3368x + 13.809 | | | | R² = 0.99586 | | | | |  | y = 3.3019x + 13.803 | | | | R² = 0.99548 | | | | |  | y = 3.3461x + 14.028 | | | | R² = 0.99755 | | | | |  | y = 3.3076x + 13.910 | | | | R² = 0.99624 | | | |  | |  | |  | |  | | |  |
|  |  | GIVa: US-Makah | | | | | | | | | | | | | | | | | | | | | | | | | | | | | | | | | | | | | |  | |  | |  | |  | | |  |
| Dil. |  | 0 h | | | | | | | | |  | 1 d | | | | | | | | |  | 1 w | | | | | | | | |  | 3 m | | | | | | | |  | | Reproducibility | | | | | | |  |
|  |  | Rpl 1 | Rpl 2 | Rpl 3 | Avrg. | | Desv | CV |  | Rpl 1 | | | Rpl 2 | Rpl 3 | Avrg. | | Desv | CV |  | Rpl 1 | | | Rpl 2 | Rpl 3 | Avrg. | | Desv | CV |  | Rpl 1 | | | Rpl 2 | Rpl 3 | Avrg. | | Desv | CV |  | | Avrg. | | Desv | | CV | |  |  |  |
| -1 |  | 20.02 | 20.66 | 20.55 | 20.41 | | 0.34 | 1.68 |  | 20.06 | | | 20.32 | 20.24 | 20.21 | | 0.13 | 0.66 |  | 20.47 | | | 20.52 | 20.35 | 20.45 | | 0.09 | 0.43 |  | 20.33 | | | 20.75 | 19.94 | 20.34 | | 0.41 | 1.99 |  | | 20.35 | | 0.25 | | 1.25 | |  |  |  |
| -2 |  | 23.95 | 23.18 | 23.24 | 23.46 | | 0.43 | 1.83 |  | 23.68 | | | 23.51 | 23.61 | 23.60 | | 0.09 | 0.36 |  | 23.77 | | | 23.64 | 23.15 | 23.52 | | 0.33 | 1.39 |  | 23.84 | | | 23.49 | 22.52 | 23.28 | | 0.68 | 2.94 |  | | 23.47 | | 0.39 | | 1.67 | |  |  |  |
| -3 |  | 27.08 | 26.77 | 26.82 | 26.89 | | 0.17 | 0.62 |  | 26.50 | | | 26.66 | 27.34 | 26.83 | | 0.45 | 1.66 |  | 26.06 | | | 26.25 | 26.13 | 26.15 | | 0.10 | 0.37 |  | 26.39 | | | 26.36 | 26.45 | 26.40 | | 0.05 | 0.17 |  | | 26.57 | | 0.38 | | 1.44 | |  |  |  |
| -4 |  | 30.91 | 30.09 | 29.53 | 30.18 | | 0.69 | 2.30 |  | 30.63 | | | 30.32 | 31.22 | 30.72 | | 0.46 | 1.49 |  | 30.36 | | | 30.13 | 30.07 | 30.19 | | 0.15 | 0.51 |  | 29.83 | | | 29.93 | 29.67 | 29.81 | | 0.13 | 0.44 |  | | 30.22 | | 0.50 | | 1.65 | |  |  |  |
| -5 |  | 33.92 | 33.99 | 33.12 | 33.68 | | 0.48 | 1.44 |  | 33.93 | | | 33.99 | 34.62 | 34.18 | | 0.38 | 1.12 |  | 33.12 | | | 33.35 | 33.62 | 33.36 | | 0.25 | 0.75 |  | 33.57 | | | 33.78 | 33.67 | 33.67 | | 0.11 | 0.31 |  | | 33.72 | | 0.42 | | 1.24 | |  |  |  |
| -6 |  | 37.12 | 37.03 | 37.18 | 37.11 | | 0.08 | 0.20 |  | 37.07 | | | 37.37 | 37.15 | 37.20 | | 0.16 | 0.42 |  | 37.12 | | | 37.66 | 37.56 | 37.45 | | 0.29 | 0.77 |  | 37.26 | | | 37.90 | 37.29 | 37.48 | | 0.36 | 0.96 |  | | 37.31 | | 0.27 | | 0.72 | |  |  |  |
| -7 |  | - | - | - | - | | - | - |  | - | | | 40.53 | - | - | | - | - |  | - | | | - | 40.61 | - | | - | - |  | - | | | - | - | - | | - | - |  | | - | | - | | - | |  |  |  |
|  |  |  |  |  |  | |  |  |  |  | | |  |  |  | |  |  |  |  | | |  |  |  | |  |  |  |  | | |  |  |  | |  |  |  | |  | |  | |  | |  |  |  |
| Rpl 1 |  | y = 3.4069x + 16.909 | | | | R² = 0. 99835 | | | | |  | y = 3.4266x + 16.652 | | | | R² = 0. 99839 | | | | |  | y = 3.3029x + 16.923 | | | | R² = 0. 99474 | | | | |  | y = 3.3509x + 16.809 | | | | R² = 0. 99683 | | | |  | |  | |  | |  | | |  |
| Rpl 2 |  | y = 3.3600x + 16.860 | | | | R² = 0. 99730 | | | | |  | y = 3.4386x + 16.660 | | | | R² = 0. 99911 | | | | |  | y = 3.3917x + 16.721 | | | | R² = 0. 99465 | | | | |  | y = 3.4340x + 16.683 | | | | R² = 0. 99319 | | | |  | |  | |  | |  | | |  |
| Rpl 3 |  | y = 3.3000x + 16.857 | | | | R² = 0. 99575 | | | | |  | y = 3.4703x + 16.884 | | | | R² = 0. 99657 | | | | |  | y = 3.4686x + 16.340 | | | | R² = 0. 99584 | | | | |  | y = 3.5263x + 15.915 | | | | R² = 0. 99709 | | | |  | |  | |  | |  | | |  |
|  |  |  |  |  |  | |  |  |  |  | | |  |  |  | |  |  |  |  | | |  |  |  | |  |  |  |  | | |  |  |  | |  |  |  | |  | |  | |  | |  |  |  |
| Avrg. |  | y = 3.3556x + 16.875 | | | | R² = 0. 99961 | | | | |  | y = 3.4451x + 16.732 | | | | R² = 0. 99907 | | | | |  | y = 3.3877x + 16.661 | | | | R² = 0. 99558 | | | | |  | y = 3.4370x + 16.469 | | | | R² = 0. 99684 | | | |  | |  | |  | |  | | |  |
|  |  | GIVb: Goby 1F | | | | | | | | | | | | | | | | | | | | | | | | | | | | | | | | | | | | | |  | |  | |  | |  | | |  |
| Dil. |  | 0 h | | | | | | | | |  | 1 d | | | | | | | | |  | 1 w | | | | | | | | |  | 3 m | | | | | | | |  | | Reproducibility | | | | | | |  |
|  |  | Rpl 1 | Rpl 2 | Rpl 3 | Avrg. | | Desv | CV |  | Rpl 1 | | | Rpl 2 | Rpl 3 | Avrg. | | Desv | CV |  | Rpl 1 | | | Rpl 2 | Rpl 3 | Avrg. | | Desv | CV |  | Rpl 1 | | | Rpl 2 | Rpl 3 | Avrg. | | Desv | CV |  | | Avrg. | | Desv | | CV | |  |  |  |
| -1 |  | 19.99 | 20.42 | 20.13 | 20.18 | | 0.22 | 1.09 |  | 20.14 | | | 20.43 | 19.29 | 19.95 | | 0.59 | 2.97 |  | 20.87 | | | 20.65 | 19.49 | 20.34 | | 0.74 | 3.65 |  | 21.19 | | | 21.31 | 21.23 | 21.24 | | 0.06 | 0.29 |  | | 20.43 | | 0.66 | | 3.23 | |  |  |  |
| -2 |  | 23.02 | 22.78 | 22.61 | 22.80 | | 0.21 | 0.90 |  | 22.01 | | | 22.92 | 22.77 | 22.57 | | 0.49 | 2.16 |  | 23.11 | | | 23.22 | 23.86 | 23.40 | | 0.41 | 1.73 |  | 23.93 | | | 24.13 | 24.91 | 24.32 | | 0.52 | 2.13 |  | | 23.27 | | 0.79 | | 3.41 | |  |  |  |
| -3 |  | 26.25 | 25.90 | 26.23 | 26.13 | | 0.20 | 0.75 |  | 25.62 | | | 25.90 | 26.46 | 25.99 | | 0.43 | 1.65 |  | 26.76 | | | 26.59 | 26.05 | 26.47 | | 0.37 | 1.40 |  | 26.99 | | | 27.65 | 27.17 | 27.27 | | 0.34 | 1.25 |  | | 26.46 | | 0.60 | | 2.25 | |  |  |  |
| -4 |  | 28.99 | 29.11 | 29.12 | 29.07 | | 0.07 | 0.25 |  | 28.80 | | | 28.92 | 29.18 | 28.97 | | 0.19 | 0.67 |  | 28.56 | | | 29.89 | 29.40 | 29.28 | | 0.67 | 2.30 |  | 30.80 | | | 31.40 | 30.01 | 30.74 | | 0.70 | 2.27 |  | | 29.52 | | 0.86 | | 2.91 | |  |  |  |
| -5 |  | 32.11 | 32.04 | 31.97 | 32.04 | | 0.07 | 0.22 |  | 31.51 | | | 31.25 | 32.87 | 31.88 | | 0.87 | 2.73 |  | 31.17 | | | 32.20 | 32.11 | 31.83 | | 0.57 | 1.79 |  | 33.67 | | | 34.37 | 32.93 | 33.66 | | 0.72 | 2.14 |  | | 32.35 | | 0.96 | | 2.96 | |  |  |  |
| -6 |  | 36.65 | 36.12 | 36.56 | 36.44 | | 0.28 | 0.78 |  | 35.31 | | | 35.65 | 35.95 | 35.64 | | 0.32 | 0.90 |  | 37.54 | | | 37.33 | 36.12 | 37.00 | | 0.77 | 2.07 |  | 37.90 | | | 38.20 | 37.50 | 37.87 | | 0.35 | 0.93 |  | | 39.38 | | 0.64 | | 1.63 | |  |  |  |
| -7 |  | - | - | 40.33 | - | | - | - |  | 39.10 | | | - | - | - | | - | - |  | - | | | - | 40.12 | - | | - | - |  | - | | | - | - | - | | - | - |  | | - | | - | | - | |  |  |  |
|  |  |  |  |  |  | |  |  |  |  | | |  |  |  | |  |  |  |  | | |  |  |  | |  |  |  |  | | |  |  |  | |  |  |  | |  | |  | |  | |  |  |  |
| Rpl 1 |  | y = 3.2374x + 16.504 | | | | R² = 0. 99383 | | | | |  | y = 3.0723x + 16.479 | | | | R² = 0. 99400 | | | | |  | y = 3.1237x + 17.069 | | | | R² = 0. 96115 | | | | |  | y = 3.3309x + 17.422 | | | | R² = 0. 99587 | | | |  | |  | |  | |  | | |  |
| Rpl 2 |  | y = 3.1283x + 16.779 | | | | R² = 0. 99476 | | | | |  | y = 2.9746x + 17.101 | | | | R² = 0. 99243 | | | | |  | y = 3.2469x + 16.949 | | | | R² = 0. 98904 | | | | |  | y = 3.3977x + 17.618 | | | | R² = 0. 99840 | | | |  | |  | |  | |  | | |  |
| Rpl 3 |  | y = 3.2320x + 16.458 | | | | R² = 0. 99298 | | | | |  | y = 3.3234x + 16.121 | | | | R² = 0. 99874 | | | | |  | y = 3.1786x + 16.713 | | | | R² = 0. 99335 | | | | |  | y = 3.0929x + 18.133 | | | | R² = 0. 98963 | | | |  | |  | |  | |  | | |  |
|  |  |  |  |  |  | |  |  |  |  | | |  |  |  | |  |  |  |  | | |  |  |  | |  |  |  |  | | |  |  |  | |  |  |  | |  | |  | |  | |  |  |  |
| Avrg. |  | y = 3.1992x + 16.580 | | | | R² = 0. 99434 | | | | |  | y = 3.1234x + 16.567 | | | | R² = 0. 99798 | | | | |  | y = 3.1830x + 16.910 | | | | R² = 0. 98787 | | | | |  | y = 3.2738x + 17.724 | | | | R² = 0. 99689 | | | |  | |  | |  | |  | | |  |
|  |  |  |  |  |  | |  |  |  |  | | |  |  |  | |  |  |  |  | | |  |  |  | |  |  |  |  | | |  |  |  | |  |  |  | |  | |  | |  | |  |  |  |
|  |  |  |  |  |  | |  |  |  |  | | |  |  |  | |  |  |  |  | | |  |  |  | |  |  |  |  | | |  |  |  | |  |  | Total Reproducibility^10^ | | | | | | | | |  |  |
|  |  |  |  |  |  | |  |  |  |  | | |  |  |  | |  |  |  |  | | |  |  |  | |  |  |  |  | | |  |  |  | |  |  |  | | Avrg. | | Desv | | CV | |  |  |  |
|  |  |  |  |  |  | |  |  |  |  | | |  |  |  | |  |  |  |  | | |  |  |  | |  |  |  |  | | |  |  |  | |  |  |  | | 19.29 | | 1.15 | | 5.96 | |  |  |  |
|  |  |  |  |  |  | |  |  |  |  | | |  |  |  | |  |  |  |  | | |  |  |  | |  |  |  |  | | |  |  |  | |  |  |  | | 22.30 | | 1.18 | | 5.29 | |  |  |  |
|  |  |  |  |  |  | |  |  |  |  | | |  |  |  | |  |  |  |  | | |  |  |  | |  |  |  |  | | |  |  |  | |  |  |  | | 25.48 | | 1.07 | | 4.20 | |  |  |  |
|  |  |  |  |  |  | |  |  |  |  | | |  |  |  | |  |  |  |  | | |  |  |  | |  |  |  |  | | |  |  |  | |  |  |  | | 28.77 | | 1.24 | | 4.31 | |  |  |  |
|  |  |  |  |  |  | |  |  |  |  | | |  |  |  | |  |  |  |  | | |  |  |  | |  |  |  |  | | |  |  |  | |  |  |  | | 32.24 | | 1.39 | | 4.31 | |  |  |  |
|  |  |  |  |  |  | |  |  |  |  | | |  |  |  | |  |  |  |  | | |  |  |  | |  |  |  |  | | |  |  |  | |  |  |  | | 35.88 | | 1.41 | | 3.93 | |  |  |  |
|  |  |  |  |  |  | |  |  |  |  | | |  |  |  | |  |  |  |  | | |  |  |  | |  |  |  |  | | |  |  |  | |  |  |  | | 39.21 | | 0.67 | | 1.71 | |  |  |  |

^1^Genogroup and reference strain; ^2^Dilution; ^3^Storage time; ^4^Replica; ^5^Average Ct; ^6^Standard deviation; ^7^Coefficient of variation; ^8^Standard curve and coefficient of determination (R^2^); ^9^Average values from the 4 Storage times; ^10^Values averaged from all replicas. storage times and genogroups.
