# Supplementary material for: Design and Evaluation of a Macroarray for Detection, Identification, and Typing of Viral Hemorrhagic Septicemia Virus (VHSV)
Source: Animals (Basel). 2021 Mar 16;11(3):841. doi: 10.3390/ani11030841 (PMC8002285; doi:10.3390/ani11030841)
Supplement: Supplementary file 1 [file animals-11-00841-s001.zip › Sppl Files/Sppl Table DOCs/Supplementary Table S1.docx]

Supplementary Table S1.- Data of RT-qPCR and regression lines for all replicas and repeats

|  |  | | ^1^G1: FR-07-21 | | | | | | | | | | | | | | | | | | | | | | | | | | | | | | | | | | | | | | | | | | | | | | | | | | | | | | | | | | | | | | | | | | | | | | | | | | |  | | |  | | | | |  | | | | |  | | | | |  | | | | | | |
| --- | --- | --- | --- | --- | --- | --- | --- | --- | --- | --- | --- | --- | --- | --- | --- | --- | --- | --- | --- | --- | --- | --- | --- | --- | --- | --- | --- | --- | --- | --- | --- | --- | --- | --- | --- | --- | --- | --- | --- | --- | --- | --- | --- | --- | --- | --- | --- | --- | --- | --- | --- | --- | --- | --- | --- | --- | --- | --- | --- | --- | --- | --- | --- | --- | --- | --- | --- | --- | --- | --- | --- | --- | --- | --- | --- | --- | --- | --- | --- | --- | --- | --- | --- | --- | --- | --- | --- | --- | --- | --- | --- | --- | --- | --- | --- | --- | --- | --- | --- | --- | --- | --- |
| ^2^Dil. |  | | ^3^0 h | | | | | | | | | | | | | | | |  | | | | 1d | | | | | | | | | | | | | | | |  | | | | 1 w | | | | | | | | | | | | | | | |  | | | | 3 m | | | | | | | | | | | | | | |  | | | ^9^Reproducibility | | | | | | | | | | | | | | |  | | | | | | |
|  |  | | ^4^Rpl 1 | | | Rpl 2 | | Rpl 3 | | ^5^Avrg. | | | | ^6^Desv | | ^7^CV | |  | | | | Rpl 1 | | | | Rpl 2 | | Rpl 3 | | Avrg. | | | Desv | | CV | |  | | | Rpl 1 | | | | | Rpl 2 | | Rpl 3 | | Avrg. | | | | Desv | | CV | |  | | | Rpl 1 | | | | | Rpl 2 | | Rpl 3 | | Avrg. | | | | Desv | | CV | |  | | | Avrg. | | | | | Desv | | | | | CV | | | |  | | |  |  |  |  |  |  |
| -1 |  | | 23.47 | | | 22.21 | | 22.40 | | 22.69 | | | | 0.68 | | 2.99 | |  | | | | 22.47 | | | | 22.21 | | 22.00 | | 22.23 | | | 0.24 | | 1.06 | |  | | | 22.43 | | | | | 24.30 | | 22.71 | | 23.15 | | | | 1.01 | | 4.36 | |  | | | 22.91 | | | | | 22.86 | | 23.13 | | 22.97 | | | | 0.14 | | 0.63 | |  | | | 22.76 | | | | | 0.64 | | | | | 2.83 | | | |  | | |  |  |  |  |  |  |
| -2 |  | | 25.26 | | | 26.10 | | 25.19 | | 25.52 | | | | 0.51 | | 1.98 | |  | | | | 25.26 | | | | 25.10 | | 25.19 | | 25.18 | | | 0.08 | | 0.32 | |  | | | 26.50 | | | | | 26.11 | | 25.70 | | 26.10 | | | | 0.40 | | 1.53 | |  | | | 26.67 | | | | | 26.63 | | 26.58 | | 26.63 | | | | 0.05 | | 0.17 | |  | | | 25.86 | | | | | 0.64 | | | | | 2.48 | | | |  | | |  |  |  |  |  |  |
| -3 |  | | 29.56 | | | 30.12 | | 29.01 | | 29.56 | | | | 0.56 | | 1.88 | |  | | | | 29.56 | | | | 29.12 | | 29.00 | | 29.23 | | | 0.29 | | 1.01 | |  | | | 29.85 | | | | | 29.93 | | 29.62 | | 29.80 | | | | 0.16 | | 0.54 | |  | | | 29.41 | | | | | 29.36 | | 29.24 | | 29.34 | | | | 0.09 | | 0.30 | |  | | | 29.48 | | | | | 0.36 | | | | | 1.23 | | | |  | | |  |  |  |  |  |  |
| -4 |  | | 32.98 | | | 33.13 | | 32.69 | | 32.93 | | | | 0.22 | | 0.68 | |  | | | | 32.98 | | | | 32.13 | | 32.69 | | 32.60 | | | 0.43 | | 1.33 | |  | | | 32.09 | | | | | 32.33 | | 32.43 | | 32.28 | | | | 0.17 | | 0.54 | |  | | | 34.04 | | | | | 33.74 | | 33.88 | | 33.89 | | | | 0.15 | | 0.44 | |  | | | 32.93 | | | | | 0.67 | | | | | 2.03 | | | |  | | |  |  |  |  |  |  |
| -5 |  | | 35.93 | | | 36.56 | | 35.45 | | 35.98 | | | | 0.56 | | 1.55 | |  | | | | 35.93 | | | | 35.56 | | 35.45 | | 35.65 | | | 0.25 | | 0.71 | |  | | | 35.88 | | | | | 37.16 | | 36.05 | | 36.36 | | | | 0.70 | | 1.91 | |  | | | 36.92 | | | | | 37.01 | | 36.85 | | 36.93 | | | | 0.08 | | 0.22 | |  | | | 36.23 | | | | | 0.64 | | | | | 1.75 | | | |  | | |  |  |  |  |  |  |
| -6 |  | | 38.76 | | | 39.16 | | 38.56 | | 38.83 | | | | 0.31 | | 0.79 | |  | | | | 38.85 | | | | 38.76 | | 38.25 | | 38.62 | | | 0.32 | | 0.84 | |  | | | 38.93 | | | | | 38.93 | | 39.07 | | 38.98 | | | | 0.08 | | 0.21 | |  | | | 39.89 | | | | | 40.12 | | 39.31 | | 39.77 | | | | 0.42 | | 1.05 | |  | | | 39.05 | | | | | 0.53 | | | | | 1.35 | | | |  | | |  |  |  |  |  |  |
| -7 |  | | 40.97 | | | - | | - | | - | | | | - | | - | |  | | | | - | | | | - | | - | | - | | | - | | - | |  | | | - | | | | | 41.08 | | - | | - | | | | - | | - | |  | | | - | | | | | - | | - | | - | | | | - | | - | |  | | | - | | | | | - | | | | | - | | | |  | | |  |  |  |  |  |  |
|  |  | |  | | |  | |  | |  | | | |  | |  | |  | | | |  | | | |  | |  | |  | | |  | |  | |  | | |  | | | | |  | |  | |  | | | |  | |  | |  | | |  | | | | |  | |  | |  | | | |  | |  | |  | | |  | | | | |  | | | | |  | | | |  | | |  |  |  |  |  |  |
| Rpl 1 |  | | y = 3.1966x + 19.805 | | | | | | | | R² = 0.99252 | | | | | | | |  | | | | y = 3.3523x + 19.109 | | | | | | | | R² = 0.99586 | | | | | | | |  | | | | y = 3.2251x + 19.659 | | | | | | | | R² = 0.99529 | | | | | | | |  | | | | y = 3.4366x + 19.612 | | | | | | | R² = 0.99561 | | | | | | | |  | | |  | | | | |  | | | | |  | | | | |  | | | | | | |
| Rpl 2 |  | | y = 3.4040x + 19.299 | | | | | | | | R² = 0.99492 | | | | | | | |  | | | | y = 3.3469x + 18.766 | | | | | | | | R² = 0.99873 | | | | | | | |  | | | | y = 3.1057x + 20.590 | | | | | | | | R² = 0.98551 | | | | | | | |  | | | | y = 3.4806x + 19.438 | | | | | | | R² = 0.99746 | | | | | | | |  | | |  | | | | |  | | | | |  | | | | |  | | | | | | |
| Rpl 3 |  | | y = 3.2931x + 19.024 | | | | | | | | R² = 0.99743 | | | | | | | |  | | | | y = 3.3063x + 18.858 | | | | | | | | R² = 0.99622 | | | | | | | |  | | | | y = 3.3046x + 19.364 | | | | | | | | R² = 0.99865 | | | | | | | |  | | | | y = 3.3243x + 19.863 | | | | | | | R² = 0.99351 | | | | | | | |  | | |  | | | | |  | | | | |  | | | | |  | | | | | | |
|  |  | |  | | |  | |  | |  | | | |  | |  | |  | | | |  | | | |  | |  | |  | | |  | |  | |  | | |  | | | | |  | |  | |  | | | |  | |  | |  | | |  | | | | |  | |  | |  | | | |  | |  | |  | | |  | | | | |  | | | | |  | | | |  | | |  |  |  |  |  |  |
| Avrg. |  | | y = 3.2979x + 19.376 | | | | | | | | R² = 0.99710 | | | | | | | |  | | | | y = 3.3351x + 18.911 | | | | | | | | R² = 0.99759 | | | | | | | |  | | | | y = 3.2118x + 19.871 | | | | | | | | R² = 0.99707 | | | | | | | |  | | | | y = 3.4138x + 19.638 | | | | | | | R² = 0.99585 | | | | | | | |  | | |  | | | | |  | | | | |  | | | | |  | | | | | | |
|  |  | | G2: DK-1p49 | | | | | | | | | | | | | | | | | | | | | | | | | | | | | | | | | | | | | | | | | | | | | | | | | | | | | | | | | | | | | | | | | | | | | | | | | | |  | | |  | | | | |  | | | | |  | | | | |  | | | | | | |
| Dil. |  | | 0 h | | | | | | | | | | | | | | | |  | | | | 1d | | | | | | | | | | | | | | | |  | | | | 1 w | | | | | | | | | | | | | | | |  | | | | 3 m | | | | | | | | | | | | | | |  | | | Reproducibility | | | | | | | | | | | | | | |  | | | | | | |
|  |  | | Rpl 1 | | | Rpl 2 | | Rpl 3 | | Avrg. | | | | Desv | | CV | |  | | | | Rpl 1 | | | | Rpl 2 | | Rpl 3 | | Avrg. | | | Desv | | CV | |  | | | Rpl 1 | | | | | Rpl 2 | | Rpl 3 | | Avrg. | | | | Desv | | CV | |  | | | Rpl 1 | | | | | Rpl 2 | | Rpl 3 | | Avrg. | | | | Desv | | CV | |  | | | Avrg. | | | | | Desv | | | | | CV | | | |  | | |  |  |  |  |  |  |
| -1 |  | | 22.95 | | | 23.09 | | 22.75 | | 22.93 | | | | 0.17 | | 0.75 | |  | | | | 22.56 | | | | 23.02 | | 23.55 | | 23.04 | | | 0.50 | | 2.15 | |  | | | 22.85 | | | | | 22.72 | | 23.25 | | 22.94 | | | | 0.28 | | 1.20 | |  | | | 22.95 | | | | | 23.09 | | 22.75 | | 22.93 | | | | 0.17 | | 0.75 | |  | | | 22.96 | | | | | 0.27 | | | | | 1.17 | | | |  | | |  |  |  |  |  |  |
| -2 |  | | 26.32 | | | 25.71 | | 26.13 | | 26.05 | | | | 0.31 | | 1.20 | |  | | | | 27.33 | | | | 27.14 | | 27.67 | | 27.38 | | | 0.27 | | 0.98 | |  | | | 27.20 | | | | | 28.70 | | 27.74 | | 27.88 | | | | 0.76 | | 2.73 | |  | | | 26.32 | | | | | 27.71 | | 27.13 | | 27.05 | | | | 0.70 | | 2.58 | |  | | | 27.09 | | | | | 0.84 | | | | | 3.11 | | | |  | | |  |  |  |  |  |  |
| -3 |  | | 29.03 | | | 30.56 | | 29.92 | | 29.84 | | | | 0.77 | | 2.58 | |  | | | | 30.70 | | | | 31.57 | | 31.72 | | 31.33 | | | 0.55 | | 1.76 | |  | | | 29.03 | | | | | 30.06 | | 28.92 | | 29.34 | | | | 0.63 | | 2.14 | |  | | | 30.03 | | | | | 30.56 | | 29.92 | | 30.17 | | | | 0.34 | | 1.13 | |  | | | 30.17 | | | | | 0.92 | | | | | 3.04 | | | |  | | |  |  |  |  |  |  |
| -4 |  | | 33.74 | | | 33.31 | | 32.22 | | 33.09 | | | | 0.78 | | 2.37 | |  | | | | 33.49 | | | | 34.92 | | 33.46 | | 33.96 | | | 0.83 | | 2.46 | |  | | | 33.74 | | | | | 33.31 | | 33.22 | | 33.42 | | | | 0.28 | | 0.83 | |  | | | 33.86 | | | | | 33.34 | | 34.22 | | 33.81 | | | | 0.44 | | 1.31 | |  | | | 33.57 | | | | | 0.64 | | | | | 1.91 | | | |  | | |  |  |  |  |  |  |
| -5 |  | | 35.62 | | | 36.86 | | 35.32 | | 35.93 | | | | 0.82 | | 2.27 | |  | | | | 35.83 | | | | 35.67 | | 35.36 | | 35.62 | | | 0.24 | | 0.67 | |  | | | 35.62 | | | | | 35.74 | | 35.27 | | 35.54 | | | | 0.24 | | 0.69 | |  | | | 36.62 | | | | | 36.86 | | 36.32 | | 36.60 | | | | 0.27 | | 0.74 | |  | | | 35.92 | | | | | 0.59 | | | | | 1.64 | | | |  | | |  |  |  |  |  |  |
| -6 |  | | 39.27 | | | 39.20 | | 39.25 | | 39.24 | | | | 0.04 | | 0.09 | |  | | | | 40.64 | | | | 39.58 | | 39.98 | | 40.07 | | | 0.54 | | 1.34 | |  | | | 39.76 | | | | | 38.37 | | 39.59 | | 39.24 | | | | 0.76 | | 1.93 | |  | | | 39.27 | | | | | 39.85 | | 40.02 | | 39.71 | | | | 0.39 | | 0.99 | |  | | | 39.57 | | | | | 0.56 | | | | | 1.42 | | | |  | | |  |  |  |  |  |  |
| -7 |  | | - | | | - | | - | | - | | | | - | | - | |  | | | | - | | | | - | | - | | - | | | - | | - | |  | | | - | | | | | 41.0 | | - | | - | | | | - | | - | |  | | | - | | | | | - | | - | | - | | | | - | | - | |  | | | - | | | | | - | | | | | - | | | |  | | |  |  |  |  |  |  |
|  |  | |  | | |  | |  | |  | | | |  | |  | |  | | | |  | | | |  | |  | |  | | |  | |  | |  | | |  | | | | |  | |  | |  | | | |  | |  | |  | | |  | | | | |  | |  | |  | | | |  | |  | |  | | |  | | | | |  | | | | |  | | | |  | | |  |  |  |  |  |  |
| Rpl 1 |  | | y = 3.2631x + 19.734 | | | | | | | | R² = 0.99283 | | | | | | | |  | | | | y = 3.3911x + 19.889 | | | | | | | | R² = 0.98747 | | | | | | | |  | | | | y = 3.272x + 19.915 | | | | | | | | R² = 0.98854 | | | | | | | |  | | | | y = 3.3237x + 19.875 | | | | | | | R² = 0.99522 | | | | | | | |  | | |  | | | | |  | | | | |  | | | | |  | | | | | | |
| Rpl 2 |  | | y = 3.3357x + 19.780 | | | | | | | | R² = 0.99136 | | | | | | | |  | | | | y = 3.1926x + 20.809 | | | | | | | | R² = 0.96995 | | | | | | | |  | | | | y = 2.9320x + 21.221 | | | | | | | | R² = 0.96777 | | | | | | | |  | | | | y = 3.2580x + 20.499 | | | | | | | R² = 0.99416 | | | | | | | |  | | |  | | | | |  | | | | |  | | | | |  | | | | | | |
| Rpl 3 |  | | y = 3.2106x + 19.695 | | | | | | | | R² = 0.99589 | | | | | | | |  | | | | y = 3.0560x + 21.261 | | | | | | | | R² = 0.97678 | | | | | | | |  | | | | y = 3.1026x + 20.473 | | | | | | | | R² = 0.98335 | | | | | | | |  | | | | y = 3.3777x + 19.905 | | | | | | | R² = 0.99298 | | | | | | | |  | | |  | | | | |  | | | | |  | | | | |  | | | | | | |
|  |  | |  | | |  | |  | |  | | | |  | |  | |  | | | |  | | | |  | |  | |  | | |  | |  | |  | | |  | | | | |  | |  | |  | | | |  | |  | |  | | |  | | | | |  | |  | |  | | | |  | |  | |  | | |  | | | | |  | | | | |  | | | |  | | |  |  |  |  |  |  |
| Avrg. |  | | y = 3.2698x + 19.736 | | | | | | | | R² = 0.99866 | | | | | | | |  | | | | y = 3.2132x + 20.653 | | | | | | | | R² = 0.98221 | | | | | | | |  | | | | y = 3.1022x + 20.536 | | | | | | | | R² = 0.98518 | | | | | | | |  | | | | y = 3.3198x + 20.093 | | | | | | | R² = 0.99671 | | | | | | | |  | | |  | | | | |  | | | | |  | | | | |  | | | | | | |
|  |  | | G3: MLA88 | | | | | | | | | | | | | | | | | | | | | | | | | | | | | | | | | | | | | | | | | | | | | | | | | | | | | | | | | | | | | | | | | | | | | | | | | | |  | | |  | | | | |  | | | | |  | | | | |  | | | | | | |
| Dil. |  | | 0 h | | | | | | | | | | | | | | | |  | | | | 1d | | | | | | | | | | | | | | | |  | | | | 1 w | | | | | | | | | | | | | | | |  | | | | 3 m | | | | | | | | | | | | | | |  | | | Reproducibility | | | | | | | | | | | | | | |  | | | | | | |
|  |  | | Rpl 1 | | | Rpl 2 | | Rpl 3 | | Avrg. | | | | Desv | | CV | |  | | | | Rpl 1 | | | | Rpl 2 | | Rpl 3 | | Avrg. | | | Desv | | CV | |  | | | Rpl 1 | | | | | Rpl 2 | | Rpl 3 | | Avrg. | | | | Desv | | CV | |  | | | Rpl 1 | | | | | Rpl 2 | | Rpl 3 | | Avrg. | | | | Desv | | CV | |  | | | Avrg. | | | | | Desv | | | | | CV | | | |  | | |  |  |  |  |  |  |
| -1 |  | | 23.28 | | | 23.92 | | 21.98 | | 23.06 | | | | 0.99 | | 4.29 | |  | | | | 23.50 | | | | 22.90 | | 23.22 | | 23.21 | | | 0.30 | | 1.29 | |  | | | 23.92 | | | | | 21.98 | | 23.50 | | 23.13 | | | | 1.02 | | 4.41 | |  | | | 23.28 | | | | | 23.92 | | 22.90 | | 23.37 | | | | 0.52 | | 2.21 | |  | | | 23.19 | | | | | 0.67 | | | | | 2.88 | | | |  | | |  |  |  |  |  |  |
| -2 |  | | 25.92 | | | 26.63 | | 25.38 | | 25.98 | | | | 0.63 | | 2.41 | |  | | | | 26.48 | | | | 26.14 | | 27.59 | | 26.74 | | | 0.76 | | 2.84 | |  | | | 26.63 | | | | | 25.38 | | 29.48 | | 27.16 | | | | 2.10 | | 7.74 | |  | | | 25.92 | | | | | 26.63 | | 26.14 | | 26.23 | | | | 0.36 | | 1.39 | |  | | | 26.53 | | | | | 1.11 | | | | | 4.18 | | | |  | | |  |  |  |  |  |  |
| -3 |  | | 29.99 | | | 30.54 | | 30.16 | | 30.23 | | | | 0.28 | | 0.93 | |  | | | | 31.89 | | | | 31.73 | | 30.36 | | 31.33 | | | 0.84 | | 2.68 | |  | | | 30.54 | | | | | 29.56 | | 31.89 | | 30.66 | | | | 1.17 | | 3.82 | |  | | | 29.99 | | | | | 30.54 | | 31.73 | | 30.75 | | | | 0.89 | | 2.89 | |  | | | 30.74 | | | | | 0.84 | | | | | 2.73 | | | |  | | |  |  |  |  |  |  |
| -4 |  | | 33.81 | | | 34.80 | | 33.67 | | 34.09 | | | | 0.62 | | 1.81 | |  | | | | 32.58 | | | | 32.26 | | 35.20 | | 33.35 | | | 1.61 | | 4.84 | |  | | | 34.80 | | | | | 35.67 | | 34.58 | | 35.02 | | | | 0.58 | | 1.65 | |  | | | 33.81 | | | | | 34.80 | | 34.26 | | 34.29 | | | | 0.50 | | 1.45 | |  | | | 34.19 | | | | | 1.02 | | | | | 2.97 | | | |  | | |  |  |  |  |  |  |
| -5 |  | | 35.93 | | | 35.41 | | 35.96 | | 35.77 | | | | 0.31 | | 0.86 | |  | | | | 36.59 | | | | 36.55 | | 36.94 | | 36.69 | | | 0.21 | | 0.58 | |  | | | 37.41 | | | | | 36.96 | | 36.59 | | 36.99 | | | | 0.41 | | 1.11 | |  | | | 36.93 | | | | | 37.41 | | 38.55 | | 37.63 | | | | 0.83 | | 2.21 | |  | | | 36.77 | | | | | 0.82 | | | | | 2.23 | | | |  | | |  |  |  |  |  |  |
| -6 |  | | 39.65 | | | 39.25 | | 39.76 | | 39.55 | | | | 0.27 | | 0.68 | |  | | | | 39.82 | | | | 39.41 | | 41.18 | | 40.14 | | | 0.93 | | 2.31 | |  | | | 39.85 | | | | | 38.52 | | 39.45 | | 39.27 | | | | 0.68 | | 1.74 | |  | | | 40.12 | | | | | 39.98 | | 39.20 | | 39.77 | | | | 0.50 | | 1.25 | |  | | | 39.68 | | | | | 0.64 | | | | | 1.61 | | | |  | | |  |  |  |  |  |  |
| -7 |  | | - | | | - | | - | | - | | | | - | | - | |  | | | | - | | | | - | | - | | - | | | - | | - | |  | | | - | | | | | 41.15 | | - | | - | | | | - | | - | |  | | | - | | | | | - | | - | | - | | | | - | | - | |  | | | - | | | | | - | | | | | - | | | |  | | |  |  |  |  |  |  |
|  |  | |  | | |  | |  | |  | | | |  | |  | |  | | | |  | | | |  | |  | |  | | |  | |  | |  | | |  | | | | |  | |  | |  | | | |  | |  | |  | | |  | | | | |  | |  | |  | | | |  | |  | |  | | |  | | | | |  | | | | |  | | | |  | | |  |  |  |  |  |  |
| Rpl 1 |  | | y = 3.3057x + 19.860 | | | | | | | | R² = 0.99426 | | | | | | | |  | | | | y = 3.2177x + 20.548 | | | | | | | | R² = 0.97902 | | | | | | | |  | | | | y = 3.3214x + 20.567 | | | | | | | | R² = 0.99124 | | | | | | | |  | | | | y = 3.4586x + 19.570 | | | | | | | R² = 0.99716 | | | | | | | |  | | |  | | | | |  | | | | |  | | | | |  | | | | | | |
| Rpl 2 |  | | y = 3.0643x + 21.033 | | | | | | | | R² = 0.97849 | | | | | | | |  | | | | y = 3.2660x + 20.067 | | | | | | | | R² = 0.97533 | | | | | | | |  | | | | y = 3.5300x + 18.990 | | | | | | | | R² = 0.95565 | | | | | | | |  | | | | y = 3.3400x + 20.523 | | | | | | | R² = 0.99214 | | | | | | | |  | | |  | | | | |  | | | | |  | | | | |  | | | | | | |
| Rpl 3 |  | | y = 3.5471x + 18.737 | | | | | | | | R² = 0.99193 | | | | | | | |  | | | | y = 3.5054x + 20.146 | | | | | | | | R² = 0.99047 | | | | | | | |  | | | | y = 2.9649x + 22.205 | | | | | | | | R² = 0.96259 | | | | | | | |  | | | | y = 3.4646x + 20.004 | | | | | | | R² = 0.96813 | | | | | | | |  | | |  | | | | |  | | | | |  | | | | |  | | | | | | |
|  |  | |  | | |  | |  | |  | | | |  | |  | |  | | | |  | | | |  | |  | |  | | |  | |  | |  | | |  | | | | |  | |  | |  | | | |  | |  | |  | | |  | | | | |  | |  | |  | | | |  | |  | |  | | |  | | | | |  | | | | |  | | | |  | | |  |  |  |  |  |  |
| Avrg. |  | | y = 3.3057x + 19.877 | | | | | | | | R² = 0.99023 | | | | | | | |  | | | | y = 3.3297x + 20.254 | | | | | | | | R² = 0.99257 | | | | | | | |  | | | | y = 3.2721x + 20.587 | | | | | | | | R² = 0.98274 | | | | | | | |  | | | | y = 3.4210x + 20.032 | | | | | | | R² = 0.99114 | | | | | | | |  | | |  | | | | |  | | | | |  | | | | |  | | | | | | |
|  |  | |  | | |  | |  | |  | | | |  | |  | |  | | | |  | | | |  | |  | |  | | |  | |  | |  | | |  | | | | |  | |  | |  | | | |  | |  | |  | | |  | | | | |  | |  | |  | | | |  | |  | |  | | |  | | | | |  | | | | |  | | | |  | | |  |  |  |  |  |  |
|  |  | | GIVa: US-Makah | | | | | | | | | | | | | | | | | | | | | | | | | | | | | | | | | | | | | | | | | | | | | | | | | | | | | | | | | | | | | | | | | | | | | | | | | | |  | | |  | | | | |  | | | | |  | | | | |  | | | | | | |
| Dil. |  | | 0 h | | | | | | | | | | | | | | | |  | | | | 1d | | | | | | | | | | | | | | | |  | | | | 1 w | | | | | | | | | | | | | | | |  | | | | 3 m | | | | | | | | | | | | | | |  | | | Reproducibility | | | | | | | | | | | | | | |  | | | | | | |
|  |  | | Rpl 1 | | | Rpl 2 | | Rpl 3 | | Avrg. | | | | Desv | | CV | |  | | | | Rpl 1 | | | | Rpl 2 | | Rpl 3 | | Avrg. | | | Desv | | CV | |  | | | Rpl 1 | | | | | Rpl 2 | | Rpl 3 | | Avrg. | | | | Desv | | CV | |  | | | Rpl 1 | | | | | Rpl 2 | | Rpl 3 | | Avrg. | | | | Desv | | CV | |  | | | Avrg. | | | | | Desv | | | | | CV | | | |  | | |  |  |  |  |  |  |
| -1 |  | | 21.96 | | | 22.73 | | 22.77 | | 22.49 | | | | 0.46 | | 2.03 | |  | | | | 22.78 | | | | 22.79 | | 22.91 | | 22.83 | | | 0.07 | | 0.32 | |  | | | 22.37 | | | | | 22.88 | | 22.56 | | 22.60 | | | | 0.26 | | 1.14 | |  | | | 23.73 | | | | | 24.88 | | 23.78 | | 24.13 | | | | 0.65 | | 2.69 | |  | | | 23.01 | | | | | 0.77 | | | | | 3.36 | | | |  | | |  |  |  |  |  |  |
| -2 |  | | 25.03 | | | 26.37 | | 26.63 | | 26.01 | | | | 0.86 | | 3.30 | |  | | | | 26.07 | | | | 26.77 | | 25.87 | | 26.24 | | | 0.47 | | 1.80 | |  | | | 25.17 | | | | | 25.48 | | 24.99 | | 25.21 | | | | 0.25 | | 0.98 | |  | | | 26.58 | | | | | 26.77 | | 27.07 | | 26.81 | | | | 0.25 | | 0.92 | |  | | | 26.07 | | | | | 0.74 | | | | | 2.85 | | | |  | | |  |  |  |  |  |  |
| -3 |  | | 29.48 | | | 29.84 | | 29.48 | | 29.60 | | | | 0.21 | | 0.70 | |  | | | | 29.18 | | | | 29.77 | | 30.41 | | 29.79 | | | 0.62 | | 2.07 | |  | | | 28.87 | | | | | 28.96 | | 29.54 | | 29.12 | | | | 0.36 | | 1.25 | |  | | | 29.84 | | | | | 31.77 | | 31.18 | | 30.93 | | | | 0.99 | | 3.20 | |  | | | 29.86 | | | | | 0.87 | | | | | 2.92 | | | |  | | |  |  |  |  |  |  |
| -4 |  | | 33.27 | | | 33.28 | | 33.29 | | 33.28 | | | | 0.01 | | 0.03 | |  | | | | 33.47 | | | | 32.84 | | 34.44 | | 33.58 | | | 0.81 | | 2.40 | |  | | | 32.56 | | | | | 31.95 | | 33.39 | | 32.63 | | | | 0.72 | | 2.21 | |  | | | 33.28 | | | | | 32.84 | | 33.47 | | 33.20 | | | | 0.32 | | 0.97 | |  | | | 33.17 | | | | | 0.60 | | | | | 1.81 | | | |  | | |  |  |  |  |  |  |
| -5 |  | | 36.54 | | | 36.85 | | 36.63 | | 36.67 | | | | 0.16 | | 0.43 | |  | | | | 36.73 | | | | 35.71 | | 34.92 | | 35.79 | | | 0.91 | | 2.54 | |  | | | 35.68 | | | | | 34.92 | | 33.88 | | 34.83 | | | | 0.90 | | 2.59 | |  | | | 36.85 | | | | | 36.71 | | 37.73 | | 37.10 | | | | 0.55 | | 1.49 | |  | | | 36.10 | | | | | 1.09 | | | | | 3.02 | | | |  | | |  |  |  |  |  |  |
| -6 |  | | 39.53 | | | 38.36 | | 39.68 | | 39.19 | | | | 0.72 | | 1.84 | |  | | | | 39.64 | | | | 39.42 | | 39.86 | | 39.64 | | | 0.22 | | 0.55 | |  | | | 40.56 | | | | | 40.02 | | 40.86 | | 40.48 | | | | 0.43 | | 1.05 | |  | | | 39.50 | | | | | 40.23 | | 40.65 | | 40.13 | | | | 0.58 | | 1.45 | |  | | | 39.86 | | | | | 0.68 | | | | | 1.70 | | | |  | | |  |  |  |  |  |  |
| -7 |  | | - | | | 40.16 | | - | | - | | | | - | | - | |  | | | | - | | | | - | | - | | - | | | - | | - | |  | | | - | | | | | - | | - | | - | | | | - | | - | |  | | | - | | | | | - | | - | | - | | | | - | | - | |  | | | - | | | | | - | | | | | - | | | |  | | |  |  |  |  |  |  |
|  |  | |  | | |  | |  | |  | | | |  | |  | |  | | | |  | | | |  | |  | |  | | |  | |  | |  | | |  | | | | |  | |  | |  | | | |  | |  | |  | | |  | | | | |  | |  | |  | | | |  | |  | |  | | |  | | | | |  | | | | |  | | | |  | | |  |  |  |  |  |  |
| Rpl 1 |  | | y = 3.6049x + 18.351^8^ | | | | | | | | R² = 0.99623 | | | | | | | |  | | | | y = 3.4449x + 19.255 | | | | | | | | R² = 0.99754 | | | | | | | |  | | | | y = 3.6049x + 18.251 | | | | | | | | R² = 0.99468 | | | | | | | |  | | | | y = 3.2314x + 20.320 | | | | | | | R² = 0.99843 | | | | | | | |  | | |  | | | | |  | | | | |  | | | | |  | | | | | | |
| Rpl 2 |  | | y = 3.2294x + 19.935 | | | | | | | | R² = 0.98962 | | | | | | | |  | | | | y = 3.2297x + 19.913 | | | | | | | | R² = 0.99754 | | | | | | | |  | | | | y = 3.3431x + 19.001 | | | | | | | | R² = 0.98952 | | | | | | | |  | | | | y = 3.0754x + 21.436 | | | | | | | R² = 0.98241 | | | | | | | |  | | |  | | | | |  | | | | |  | | | | |  | | | | | | |
| Rpl 3 |  | | y = 3.3817x + 19.577 | | | | | | | | R² = 0.99866 | | | | | | | |  | | | | y = 3.3123x + 19.809 | | | | | | | | R² = 0.97537 | | | | | | | |  | | | | y = 3.4863x + 18.668 | | | | | | | | R² = 0.96414 | | | | | | | |  | | | | y = 3.3891x + 20.451 | | | | | | | R² = 0.99622 | | | | | | | |  | | |  | | | | |  | | | | |  | | | | |  | | | | | | |
|  |  | |  | | |  | |  | |  | | | |  | |  | |  | | | |  | | | |  | |  | |  | | |  | |  | |  | | |  | | | | |  | |  | |  | | | |  | |  | |  | | |  | | | | |  | |  | |  | | | |  | |  | |  | | |  | | | | |  | | | | |  | | | |  | | |  |  |  |  |  |  |
| Avrg. |  | | y = 3.4053x + 19.288 | | | | | | | | R² = 0.99702 | | | | | | | |  | | | | y = 3.3290x + 19.659 | | | | | | | | R² = 0.99645 | | | | | | | |  | | | | y = 3.4781x + 18.640 | | | | | | | | R² = 0.98702 | | | | | | | |  | | | | y = 3.2320x + 20.736 | | | | | | | R² = 0.99625 | | | | | | | |  | | |  | | | | |  | | | | |  | | | | |  | | | | | | |
|  |  | | GIVb: Goby 1F | | | | | | | | | | | | | | | | | | | | | | | | | | | | | | | | | | | | | | | | | | | | | | | | | | | | | | | | | | | | | | | | | | | | | | | | | | |  | | |  | | | | |  | | | | |  | | | | |  | | | | | | |
| Dil. |  | | 0 h | | | | | | | | | | | | | | | |  | | | | 1d | | | | | | | | | | | | | | | |  | | | | 1 w | | | | | | | | | | | | | | | |  | | | | 3 m | | | | | | | | | | | | | | |  | | | Reproducibility | | | | | | | | | | | | | | |  | | | | | | |
|  |  | | Rpl 1 | | | Rpl 2 | | Rpl 3 | | Avrg. | | | | Desv | | CV | |  | | | | Rpl 1 | | | | Rpl 2 | | Rpl 3 | | Avrg. | | | Desv | | CV | |  | | | Rpl 1 | | | | | Rpl 2 | | Rpl 3 | | Avrg. | | | | Desv | | CV | |  | | | Rpl 1 | | | | | Rpl 2 | | Rpl 3 | | Avrg. | | | | Desv | | CV | |  | | | Avrg. | | | | | Desv | | | | | CV | | | |  | | |  |  |  |  |  |  |
| -1 |  | | 22.86 | | | 22.73 | | 23.77 | | 23.12 | | | | 0.57 | | 2.45 | |  | | | | 22.85 | | | | 24.88 | | 22.47 | | 23.40 | | | 1.30 | | 5.54 | |  | | | 22.78 | | | | | 22.39 | | 23.91 | | 23.03 | | | | 0.79 | | 3.43 | |  | | | 22.85 | | | | | 22.72 | | 23.25 | | 22.94 | | | | 0.28 | | 1.20 | |  | | | 23.12 | | | | | 0.72 | | | | | 3.13 | | | |  | | |  |  |  |  |  |  |
| -2 |  | | 26.63 | | | 26.77 | | 26.63 | | 26.68 | | | | 0.08 | | 0.30 | |  | | | | 26.65 | | | | 26.35 | | 27.55 | | 26.85 | | | 0.62 | | 2.33 | |  | | | 26.07 | | | | | 26.77 | | 25.87 | | 26.24 | | | | 0.47 | | 1.80 | |  | | | 27.20 | | | | | 28.40 | | 27.74 | | 27.78 | | | | 0.60 | | 2.16 | |  | | | 26.89 | | | | | 0.72 | | | | | 2.69 | | | |  | | |  |  |  |  |  |  |
| -3 |  | | 30.48 | | | 29.84 | | 30.48 | | 30.27 | | | | 0.37 | | 1.22 | |  | | | | 30.08 | | | | 29.81 | | 29.54 | | 29.81 | | | 0.27 | | 0.91 | |  | | | 29.18 | | | | | 29.77 | | 29.41 | | 29.45 | | | | 0.30 | | 1.01 | |  | | | 30.70 | | | | | 31.57 | | 31.72 | | 31.33 | | | | 0.55 | | 1.76 | |  | | | 30.22 | | | | | 0.81 | | | | | 2.67 | | | |  | | |  |  |  |  |  |  |
| -4 |  | | 32.74 | | | 33.28 | | 32.29 | | 32.77 | | | | 0.50 | | 1.51 | |  | | | | 33.52 | | | | 32.94 | | 32.51 | | 32.99 | | | 0.51 | | 1.54 | |  | | | 33.47 | | | | | 32.84 | | 33.44 | | 33.25 | | | | 0.36 | | 1.07 | |  | | | 33.74 | | | | | 34.92 | | 33.46 | | 34.04 | | | | 0.77 | | 2.28 | |  | | | 33.26 | | | | | 0.69 | | | | | 2.07 | | | |  | | |  |  |  |  |  |  |
| -5 |  | | 35.91 | | | 36.85 | | 36.63 | | 36.46 | | | | 0.49 | | 1.35 | |  | | | | 36.45 | | | | 35.12 | | 36.92 | | 36.16 | | | 0.93 | | 2.58 | |  | | | 36.85 | | | | | 35.74 | | 35.92 | | 36.17 | | | | 0.60 | | 1.65 | |  | | | 35.62 | | | | | 35.81 | | 35.27 | | 35.57 | | | | 0.27 | | 0.77 | |  | | | 36.09 | | | | | 0.63 | | | | | 1.74 | | | |  | | |  |  |  |  |  |  |
| -6 |  | | 38.53 | | | 38.36 | | 39.68 | | 38.86 | | | | 0.72 | | 1.85 | |  | | | | 38.91 | | | | 38.71 | | 39.73 | | 39.12 | | | 0.54 | | 1.38 | |  | | | 39.74 | | | | | 39.28 | | 39.29 | | 39.44 | | | | 0.26 | | 0.67 | |  | | | 39.96 | | | | | 39.84 | | 40.48 | | 40.09 | | | | 0.34 | | 0.85 | |  | | | 39.38 | | | | | 0.64 | | | | | 1.63 | | | |  | | |  |  |  |  |  |  |
| -7 |  | | 40.57 | | | 41.21 | | - | | - | | | | - | | - | |  | | | | 41.19 | | | | 40.87 | | - | | - | | | - | | - | |  | | | - | | | | | - | | - | | - | | | | - | | - | |  | | | - | | | | | - | | - | | - | | | | - | | - | |  | | | - | | | | | - | | | | | - | | | |  | | |  |  |  |  |  |  |
|  |  | |  | | |  | |  | |  | | | |  | |  | |  | | | |  | | | |  | |  | |  | | |  | |  | |  | | |  | | | | |  | |  | |  | | | |  | |  | |  | | |  | | | | |  | |  | |  | | | |  | |  | |  | | |  | | | | |  | | | | |  | | | |  | | |  |  |  |  |  |  |
| Rpl 1 |  | | y = 3.0986x + 20.347 | | | | | | | | R² = 0.99277 | | | | | | | |  | | | | y = 3.2326x + 20.096 | | | | | | | | R² = 0.9949 | | | | | | | |  | | | | y = 3.4694x + 19.205 | | | | | | | | R² = 0.99753 | | | | | | | |  | | | | y = 3.2529x + 20.293 | | | | | | | R² = 0.98849 | | | | | | | |  | | |  | | | | |  | | | | |  | | | | |  | | | | | | |
| Rpl 2 |  | | y = 3.1951x + 20.122 | | | | | | | | R² = 0.98889 | | | | | | | |  | | | | y = 2.8169x + 21.443 | | | | | | | | R² = 0.9909 | | | | | | | |  | | | | y = 3.2694x + 19.689 | | | | | | | | R² = 0.99582 | | | | | | | |  | | | | y = 3.1766x + 21.092 | | | | | | | R² = 0.96228 | | | | | | | |  | | |  | | | | |  | | | | |  | | | | |  | | | | | | |
| Rpl 3 |  | | y = 3.1817x + 20.444 | | | | | | | | R² = 0.99345 | | | | | | | |  | | | | y = 3.3537x + 19.715 | | | | | | | | R² = 0.9886 | | | | | | | |  | | | | y = 3.1737x + 20.199 | | | | | | | | R² = 0.99344 | | | | | | | |  | | | | y = 3.1566x + 20.939 | | | | | | | R² = 0.97129 | | | | | | | |  | | |  | | | | |  | | | | |  | | | | |  | | | | | | |
|  |  | |  | | |  | |  | |  | | | |  | |  | |  | | | |  | | | |  | |  | |  | | |  | |  | |  | | |  | | | | |  | |  | |  | | | |  | |  | |  | | |  | | | | |  | |  | |  | | | |  | |  | |  | | |  | | | | |  | | | | |  | | | |  | | |  |  |  |  |  |  |
| Avrg. |  | | y = 3.1585x + 20.304 | | | | | | | | R² = 0.99612 | | | | | | | |  | | | | y = 3.1344x + 20.418 | | | | | | | | R² = 0.9996 | | | | | | | |  | | | | y = 3.3042x + 19.698 | | | | | | | | R² = 0.9992 | | | | | | | |  | | | | y = 3.1668x + 20.930 | | | | | | | R² = 0.97486 | | | | | | | |  | | |  | | | | |  | | | | |  | | | | |  | | | | | | |
|  | |  | |  |  | |  | |  | | | |  | |  | |  | | | |  | | | |  | |  | |  | | | | |  | |  | |  | | | |  | | | |  | |  | |  | | | |  | |  | |  | | | |  | | | |  | |  | | |  | | |  | |  | | |  | | | | |  | | | | |  | | | | | |  | | | | | | |  |
|  | |  | |  |  | |  | |  | | | |  | |  | |  | | | |  | | | |  | |  | |  | | | | |  | |  | |  | | | |  | | | |  | |  | |  | | | |  | |  | |  | | | |  | | | |  | |  | | |  | | |  | |  | | | Total Reproducibility^10^ | | | | | | | | | | | | | | | | | | | | | | | |
|  | |  | |  |  | |  | |  | | | |  | |  | |  | | | |  | | | |  | |  | |  | | | | |  | |  | |  | | | |  | | | |  | |  | |  | | | |  | |  | |  | | | |  | | | |  | |  | | |  | | |  | |  | | |  | | | Avrg. | | | | | Desv | | | | | CV | | | | | |  | |  |  |  |
|  | |  | |  |  | |  | |  | | | |  | |  | |  | | | |  | | | |  | |  | |  | | | | |  | |  | |  | | | |  | | | |  | |  | |  | | | |  | |  | |  | | | |  | | | |  | |  | | |  | | |  | |  | | |  | | | 23.01 | | | | | 0.64 | | | | | 2.77 | | | | | |  | |  |  |  |
|  | |  | |  |  | |  | |  | | | |  | |  | |  | | | |  | | | |  | |  | |  | | | | |  | |  | |  | | | |  | | | |  | |  | |  | | | |  | |  | |  | | | |  | | | |  | |  | | |  | | |  | |  | | |  | | | 26.49 | | | | | 0.93 | | | | | 3.51 | | | | | |  | |  |  |  |
|  | |  | |  |  | |  | |  | | | |  | |  | |  | | | |  | | | |  | |  | |  | | | | |  | |  | |  | | | |  | | | |  | |  | |  | | | |  | |  | |  | | | |  | | | |  | |  | | |  | | |  | |  | | |  | | | 30.09 | | | | | 0.87 | | | | | 2.88 | | | | | |  | |  |  |  |
|  | |  | |  |  | |  | |  | | | |  | |  | |  | | | |  | | | |  | |  | |  | | | | |  | |  | |  | | | |  | | | |  | |  | |  | | | |  | |  | |  | | | |  | | | |  | |  | | |  | | |  | |  | | |  | | | 33.42 | | | | | 0.84 | | | | | 2.50 | | | | | |  | |  |  |  |
|  | |  | |  |  | |  | |  | | | |  | |  | |  | | | |  | | | |  | |  | |  | | | | |  | |  | |  | | | |  | | | |  | |  | |  | | | |  | |  | |  | | | |  | | | |  | |  | | |  | | |  | |  | | |  | | | 36.22 | | | | | 0.80 | | | | | 2.22 | | | | | |  | |  |  |  |
|  | |  | |  |  | |  | |  | | | |  | |  | |  | | | |  | | | |  | |  | |  | | | | |  | |  | |  | | | |  | | | |  | |  | |  | | | |  | |  | |  | | | |  | | | |  | |  | | |  | | |  | |  | | |  | | | 39.51 | | | | | 0.65 | | | | | 1.66 | | | | | |  | |  |  |  |
|  | |  | |  | | | | | | | |  | | | | | | | |  | | | |  | | | | | | | |  | | | | | | | | |  | | |  | | | | | | | |  | | | | | | | | |  | | |  | | | | | | | |  | | | | | | | | | |  |  | | | | |  | | | | |  | | | | | |  | |  |  |
|  | |  | |  | | | | | | | |  | | | | | | | |  | | | |  | | | | | | | |  | | | | | | | | |  | | |  | | | | | | | |  | | | | | | | | |  | | |  | | | | | | | |  | | | | | | | | | |  |  | | | | |  | | | | |  | | | | | |  | |  |  |
|  | |  | |  | | | | | | | |  | | | | | | | |  | | | |  | | | | | | | |  | | | | | | | | |  | | |  | | | | | | | |  | | | | | | | | |  | | |  | | | | | | | |  | | | | | | | | | |  |  | | | | |  | | | | |  | | | | | |  | |  |  |
|  | |  | |  | | | | | | | |  | | | | | | | |  | | | |  | | | | | | | |  | | | | | | | | |  | | |  | | | | | | | |  | | | | | | | | |  | | |  | | | | | | | |  | | | | | | | | | |  |  | | | | |  | | | | |  | | | | | |  | |  |  |
|  | |  | |  | | | | | | | |  | | | | | | | |  | | | |  | | | | | | | |  | | | | | | | | |  | | |  | | | | | | | |  | | | | | | | | |  | | |  | | | | | | | |  | | | | | | | | | |  |  | | | | |  | | | | |  | | | | | |  | |  |  |
|  | |  | |  | | | | | | | |  | | | | | | | |  | | | |  | | | | | | | |  | | | | | | | | |  | | |  | | | | | | | |  | | | | | | | | |  | | |  | | | | | | | |  | | | | | | | | | |  |  | | | | |  | | | | |  | | | | | |  | |  |  |
|  | |  | |  | | | | | | | |  | | | | | | | |  | | | |  | | | | | | | |  | | | | | | | | |  | | |  | | | | | | | |  | | | | | | | | |  | | |  | | | | | | | |  | | | | | | | | | |  |  | | | | |  | | | | |  | | | | | |  | |  |  |
|  | |  | |  | | | | | | | |  | | | | | | | |  | | | |  | | | | | | | |  | | | | | | | | |  | | |  | | | | | | | |  | | | | | | | | |  | | |  | | | | | | | |  | | | | | | | | | |  |  | | | | |  | | | | |  | | | | | |  | |  |  |

^1^Genogroup and reference strain; ^2^Dilution; ^3^Storage time; ^4^Replica; ^5^Average Ct; ^6^Standard deviation; ^7^Coefficient of variation; ^8^Standar curve and coefficient of determination (R^2^); ^9^Average values from the 4 Storage times; ^10^Values averaged from all replicas. storage times and genogroups.
