# Supplementary material for: Design and Evaluation of a Macroarray for Detection, Identification, and Typing of Viral Hemorrhagic Septicemia Virus (VHSV)
Source: Animals (Basel). 2021 Mar 16;11(3):841. doi: 10.3390/ani11030841 (PMC8002285; doi:10.3390/ani11030841)
Supplement: Supplementary file 1 [file animals-11-00841-s001.zip › Sppl Files/Sppl Table PDF/Supplementary Table S2.pdf]

Supplementary Table S2.- Data of qPCR and regression lines for all replicas and repeats

[1]G1: FR-07-21

[2]Dil.	[3]0 h						1 d						1 w						3 m						Reproducibility[9]		
	[4]Rpl 1	Rpl 2	Rpl 3	[5]Avrg.	[6]Desv	[7]CV	Rpl 1	Rpl 2	Rpl 3	Avrg.	Desv	CV	Rpl 1	Rpl 2	Rpl 3	Avrg.	Desv	CV	Rpl 1	Rpl 2	Rpl 3	Avrg.	Desv	CV	Avrg.	Desv	CV
-1	19.29	18.97	19.31	19.19	0.19	0.99	19.27	19.26	18.76	19.10	0.29	1.53	19.34	18.37	18.55	18.75	0.52	2.75	19.19	18.52	18.23	18.65	0.49	2.64	18.92	0.41	2.18
-2	22.32	21.50	22.15	21.99	0.43	1.97	21.69	21.32	21.96	21.66	0.32	1.48	22.79	21.20	21.36	21.78	0.88	4.02	21.26	22.61	21.23	21.70	0.79	3.63	21.78	0.57	2.61
-3	25.16	25.24	25.22	25.20	0.04	0.17	25.45	25.24	25.38	25.36	0.11	0.42	25.63	25.03	25.17	25.28	0.31	1.24	23.82	25.13	25.11	24.69	0.75	3.04	25.13	0.44	1.77
-4	28.12	28.57	28.59	28.43	0.27	0.95	28.83	28.52	28.29	28.55	0.27	0.95	28.95	28.73	28.67	28.78	0.15	0.51	28.03	28.47	28.67	28.39	0.33	1.15	28.54	0.28	0.96
-5	32.80	32.22	32.42	32.48	0.30	0.91	32.98	33.13	33.21	33.11	0.12	0.35	32.66	32.08	32.76	32.50	0.37	1.13	32.57	33.01	32.99	32.86	0.25	0.76	32.74	0.36	1.10
-6	35.09	34.94	35.87	35.30	0.50	1.41	35.69	35.92	36.03	35.88	0.17	0.48	35.83	36.32	36.21	36.12	0.26	0.71	34.65	34.90	35.01	34.85	0.18	0.53	35.54	0.58	1.63
-7	38.26	38.56	39.43	38.75	0.61	1.57	38.24	38.56	38.87	38.56	0.32	0.82	38.03	38.75	39.85	38.88	0.92	2.36	38.57	39.37	40.73	39.56	1.09	2.76	38.93	0.78	2.01
-8	-	-	-	-	-	-	-	-	41.09	-	-	-	-	40.81	-	-	-	-	-	-	-	-	-	-	-	-	-
Rpl 1	y = 3.2171x + 15.849[8]				R^2 = 0.99554		y = 3.3014x + 15.673				R^2 = 0.99535		y = 3.1850x + 16.293				R^2 = 0.99727		y = 3.3454x + 14.917				R^2 = 0.98937				
Rpl 2	y = 3.3079x + 15.338				R^2 = 0.99839		y = 3.3925x + 15.280				R^2 = 0.99339		y = 3.5154x + 14.579				R^2 = 0.99747		y = 3.3932x + 15.286				R^2 = 0.99439				
Rpl 3	y = 3.3927x + 15.425				R^2 = 0.99806		y = 3.4393x + 15.171				R^2 = 0.99594		y = 3.6139x + 14.483				R^2 = 0.99870		y = 3.6764x + 14.147				R^2 = 0.99308				
Avrg.	y = 3.3059x + 15.537				R^2 = 0.99836		y = 3.3777x + 15.375				R^2 = 0.99544		y = 3.4381x + 15.118				R^2 = 0.99883		y = 3.4717x + 14.783				R^2 = 0.99468				

G2: DK-1p49

Dil.	0 h						1 d						1 w						3 m						Reproducibility		
	Rpl 1	Rpl 2	Rpl 3	Avrg.	Desv	CV	Rpl 1	Rpl 2	Rpl 3	Avrg.	Desv	CV	Rpl 1	Rpl 2	Rpl 3	Avrg.	Desv	CV	Rpl 1	Rpl 2	Rpl 3	Avrg.	Desv	CV	Avrg.	Desv	CV
-1	19.24	19.05	19.11	19.13	0.10	0.51	19.14	18.98	19.06	19.06	0.08	0.42	18.93	19.09	19.23	19.08	0.15	0.79	19.86	19.28	19.54	19.56	0.29	1.49	19.21	0.26	1.36
-2	22.01	22.03	22.77	22.27	0.43	1.94	22.01	22.03	22.36	22.13	0.20	0.89	22.35	22.47	22.81	22.54	0.24	1.06	22.61	22.77	22.86	22.75	0.13	0.56	22.42	0.34	1.52
-3	25.41	25.50	25.33	25.41	0.09	0.33	25.80	25.60	24.73	25.38	0.57	2.24	25.29	25.25	24.96	25.17	0.18	0.72	25.51	25.39	25.40	25.43	0.07	0.26	25.35	0.28	1.11
-4	28.68	29.12	28.46	28.75	0.34	1.17	28.86	29.09	28.95	28.97	0.12	0.40	28.90	28.19	28.39	28.49	0.37	1.28	29.01	28.69	28.77	28.82	0.17	0.58	28.76	0.29	1.01
-5	31.86	32.09	31.95	31.97	0.12	0.36	32.06	32.28	32.84	32.39	0.40	1.24	33.14	32.03	33.01	32.73	0.61	1.85	33.03	32.69	32.97	32.90	0.18	0.55	32.50	0.49	1.52
-6	35.22	36.00	35.27	35.50	0.44	1.23	36.16	35.23	36.84	36.08	0.81	2.24	36.04	36.51	35.94	36.16	0.30	0.84	36.23	36.69	36.28	36.40	0.25	0.69	36.03	0.55	1.53
-7	39.19	40.24	38.94	39.46	0.69	1.75	39.87	39.45	39.32	39.55	0.29	0.73	39.93	39.73	39.48	39.71	0.23	0.57	39.64	38.96	39.16	39.25	0.35	0.89	39.49	0.40	1.02
-8	-	-	40.93	-	-	-	-	-	-	-	-	-	-	-	-	-	-	-	-	-	-	-	-	-	-	-	-
Rpl 1	y = 3.3114x + 15.556				R^2 = 0.99837		y = 3.4554x + 15.307				R^2 = 0.99790		y = 3.5082x + 15.193				R^2 = 0.99826		y = 3.3607x + 15.970				R^2 = 0.99735				
Rpl 2	y = 3.5036x + 15.133				R^2 = 0.99744		y = 3.3746x + 15.453				R^2 = 0.99863		y = 3.4564x + 15.213				R^2 = 0.99477		y = 3.3636x + 15.756				R^2 = 0.99596				
Rpl 3	y = 3.2539x + 15.817				R^2 = 0.99817		y = 3.4946x + 15.179				R^2 = 0.99500		y = 3.3950x + 15.537				R^2 = 0.99472		y = 3.3311x + 15.959				R^2 = 0.99698				
Avrg.	y = 3.3563x + 15.502				R^2 = 0.99852		y = 3.4415x + 15.313				R^2 = 0 99931		y = 3.4532x + 15.314				R^2 = 0.99716		y = 3.3518x + 15.895				R^2 = 0.99733				

G3: MLA88

Dil.	0 h						1 d						1 w						3 m						Reproducibility		
	Rpl 1	Rpl 2	Rpl 3	Avrg.	Desv	CV	Rpl 1	Rpl 2	Rpl 3	Avrg.	Desv	CV	Rpl 1	Rpl 2	Rpl 3	Avrg.	Desv	CV	Rpl 1	Rpl 2	Rpl 3	Avrg.	Desv	CV	Avrg.	Desv	CV
-1	17.26	17.27	17.21	17.25	0.03	0.19	17.28	17.26	17.38	17.31	0.06	0.37	17.32	17.82	18.07	17.74	0.38	2.15	18.01	17.59	17.74	17.78	0.21	1.20	17.52	0.32	1.80
-2	20.46	20.55	20.96	20.66	0.27	1.29	20.88	20.45	20.21	20.51	0.34	1.65	20.10	21.05	21.28	20.81	0.63	3.01	20.50	20.17	20.29	20.32	0.17	0.82	20.58	0.38	1.86
-3	24.20	24.07	24.25	24.17	0.09	0.38	24.21	24.20	23.58	24.00	0.36	1.50	23.06	24.04	24.45	23.85	0.71	2.99	24.11	23.46	23.24	23.60	0.45	1.92	23.91	0.45	1.88
-4	26.64	26.90	26.53	26.69	0.19	0.71	26.68	26.39	26.39	26.49	0.17	0.63	26.98	27.19	27.21	27.13	0.13	0.47	27.52	26.76	26.88	27.05	0.41	1.51	26.84	0.35	1.29
-5	29.96	29.52	29.71	29.73	0.22	0.74	29.84	29.79	28.82	29.48	0.58	1.95	30.63	30.32	30.22	30.39	0.21	0.70	30.21	30.14	29.65	30.00	0.31	1.02	29.90	0.47	1.56
-6	33.39	34.91	33.26	33.85	0.92	2.71	35.77	34.42	31.65	33.95	2.10	6.19	33.01	34.88	33.93	33.94	0.94	2.75	33.58	33.37	33.50	33.48	0.11	0.32	33.81	1.07	3.18
-7	37.99	38.14	37.09	37.74	0.57	1.50	39.02	37.42	35.58	37.34	1.72	4.61	38.64	37.96	37.50	38.03	0.57	1.51	36.11	37.24	39.88	37.74	1.93	5.13	37.71	1.18	3.14
-8	-	41.13	40.31	-	-	-	-	41.05	40.73	-	-	-	-	41.01	40.97	-	-	-	40.11	-	-	-	-	-	-	-	-

	0 h		1 d		1 w		3 m	
Rpl 1	y = 3.3504x + 13.727	R² = 0.99503	y = 3.5939x + 13.293	R² = 0.98773	y = 3.4768x + 13.199	R² = 0.99098	y = 3.0914x + 14.783	R² = 0.99800
Rpl 2	y = 3.4564x + 13.511	R² = 0.99235	y = 3.3575x + 13.703	R² = 0.99484	y = 3.3700x + 14.129	R² = 0.99647	y = 3.2868x + 13.814	R² = 0.99806
Rpl 3	y = 3.2036x + 14.187	R² = 0.99623	y = 2.9543x + 14.413	R² = 0.99698	y = 3.1914x + 14.757	R² = 0.99806	y = 3.5446x + 13.133	R² = 0.97884
Avrg.	y = 3.3368x + 13.809	R² = 0.99586	y = 3.3019x + 13.803	R² = 0.99548	y = 3.3461x + 14.028	R² = 0.99755	y = 3.3076x + 13.910	R² = 0.99624

GIVa: US-Makah

Dil.	0 h						1 d						1 w						3 m						Reproducibility		
	Rpl 1	Rpl 2	Rpl 3	Avrg.	Desv	CV	Rpl 1	Rpl 2	Rpl 3	Avrg.	Desv	CV	Rpl 1	Rpl 2	Rpl 3	Avrg.	Desv	CV	Rpl 1	Rpl 2	Rpl 3	Avrg.	Desv	CV	Avrg.	Desv	CV
-1	20.02	20.66	20.55	20.41	0.34	1.68	20.06	20.32	20.24	20.21	0.13	0.66	20.47	20.52	20.35	20.45	0.09	0.43	20.33	20.75	19.94	20.34	0.41	1.99	20.35	0.25	1.25
-2	23.95	23.18	23.24	23.46	0.43	1.83	23.68	23.51	23.61	23.60	0.09	0.36	23.77	23.64	23.15	23.52	0.33	1.39	23.84	23.49	22.52	23.28	0.68	2.94	23.47	0.39	1.67
-3	27.08	26.77	26.82	26.89	0.17	0.62	26.50	26.66	27.34	26.83	0.45	1.66	26.06	26.25	26.13	26.15	0.10	0.37	26.39	26.36	26.45	26.40	0.05	0.17	26.57	0.38	1.44
-4	30.91	30.09	29.53	30.18	0.69	2.30	30.63	30.32	31.22	30.72	0.46	1.49	30.36	30.13	30.07	30.19	0.15	0.51	29.83	29.93	29.67	29.81	0.13	0.44	30.22	0.50	1.65
-5	33.92	33.99	33.12	33.68	0.48	1.44	33.93	33.99	34.62	34.18	0.38	1.12	33.12	33.35	33.62	33.36	0.25	0.75	33.57	33.78	33.67	33.67	0.11	0.31	33.72	0.42	1.24
-6	37.12	37.03	37.18	37.11	0.08	0.20	37.07	37.37	37.15	37.20	0.16	0.42	37.12	37.66	37.56	37.45	0.29	0.77	37.26	37.90	37.29	37.48	0.36	0.96	37.31	0.27	0.72
-7	-	-	-	-	-	-	-	40.53	-	-	-	-	-	-	40.61	-	-	-	-	-	-	-	-	-	-	-	-

	0 h		1 d		1 w		3 m	
Rpl 1	y = 3.4069x + 16.909	R² = 0. 99835	y = 3.4266x + 16.652	R² = 0. 99839	y = 3.3029x + 16.923	R² = 0. 99474	y = 3.3509x + 16.809	R² = 0. 99683
Rpl 2	y = 3.3600x + 16.860	R² = 0. 99730	y = 3.4386x + 16.660	R² = 0. 99911	y = 3.3917x + 16.721	R² = 0. 99465	y = 3.4340x + 16.683	R² = 0. 99319
Rpl 3	y = 3.3000x + 16.857	R² = 0. 99575	y = 3.4703x + 16.884	R² = 0. 99657	y = 3.4686x + 16.340	R² = 0. 99584	y = 3.5263x + 15.915	R² = 0. 99709
Avrg.	y = 3.3556x + 16.875	R² = 0. 99961	y = 3.4451x + 16.732	R² = 0. 99907	y = 3.3877x + 16.661	R² = 0. 99558	y = 3.4370x + 16.469	R² = 0. 99684

GIVb: Goby 1F

Dil.	0 h						1 d						1 w						3 m						Reproducibility		
	Rpl 1	Rpl 2	Rpl 3	Avrg.	Desv	CV	Rpl 1	Rpl 2	Rpl 3	Avrg.	Desv	CV	Rpl 1	Rpl 2	Rpl 3	Avrg.	Desv	CV	Rpl 1	Rpl 2	Rpl 3	Avrg.	Desv	CV	Avrg.	Desv	CV
-1	19.99	20.42	20.13	20.18	0.22	1.09	20.14	20.43	19.29	19.95	0.59	2.97	20.87	20.65	19.49	20.34	0.74	3.65	21.19	21.31	21.23	21.24	0.06	0.29	20.43	0.66	3.23
-2	23.02	22.78	22.61	22.80	0.21	0.90	22.01	22.92	22.77	22.57	0.49	2.16	23.11	23.22	23.86	23.40	0.41	1.73	23.93	24.13	24.91	24.32	0.52	2.13	23.27	0.79	3.41
-3	26.25	25.90	26.23	26.13	0.20	0.75	25.62	25.90	26.46	25.99	0.43	1.65	26.76	26.59	26.05	26.47	0.37	1.40	26.99	27.65	27.17	27.27	0.34	1.25	26.46	0.60	2.25
-4	28.99	29.11	29.12	29.07	0.07	0.25	28.80	28.92	29.18	28.97	0.19	0.67	28.56	29.89	29.40	29.28	0.67	2.30	30.80	31.40	30.01	30.74	0.70	2.27	29.52	0.86	2.91
-5	32.11	32.04	31.97	32.04	0.07	0.22	31.51	31.25	32.87	31.88	0.87	2.73	31.17	32.20	32.11	31.83	0.57	1.79	33.67	34.37	32.93	33.66	0.72	2.14	32.35	0.96	2.96
-6	36.65	36.12	36.56	36.44	0.28	0.78	35.31	35.65	35.95	35.64	0.32	0.90	37.54	37.33	36.12	37.00	0.77	2.07	37.90	38.20	37.50	37.87	0.35	0.93	39.38	0.64	1.63
-7	-	-	40.33	-	-	-	39.10	-	-	-	-	-	-	-	40.12	-	-	-	-	-	-	-	-	-	-	-	-

	0 h		1 d		1 w		3 m	
Rpl 1	y = 3.2374x + 16.504	R² = 0. 99383	y = 3.0723x + 16.479	R² = 0. 99400	y = 3.1237x + 17.069	R² = 0. 96115	y = 3.3309x + 17.422	R² = 0. 99587
Rpl 2	y = 3.1283x + 16.779	R² = 0. 99476	y = 2.9746x + 17.101	R² = 0. 99243	y = 3.2469x + 16.949	R² = 0. 98904	y = 3.3977x + 17.618	R² = 0. 99840
Rpl 3	y = 3.2320x + 16.458	R² = 0. 99298	y = 3.3234x + 16.121	R² = 0. 99874	y = 3.1786x + 16.713	R² = 0. 99335	y = 3.0929x + 18.133	R² = 0. 98963
Avrg.	y = 3.1992x + 16.580	R² = 0. 99434	y = 3.1234x + 16.567	R² = 0. 99798	y = 3.1830x + 16.910	R² = 0. 98787	y = 3.2738x + 17.724	R² = 0. 99689

Total Reproducibility[10]		
Avrg.	Desv	CV
19.29	1.15	5.96
22.30	1.18	5.29
25.48	1.07	4.20
28.77	1.24	4.31
32.24	1.39	4.31
35.88	1.41	3.93
39.21	0.67	1.71

[1]Genogroup and reference strain; [2]Dilution; [3]Storage time; [4]Replica; [5]Average Ct; [6]Standard deviation; [7]Coefficient of variation; [8]Standar curve and coefficient of determination; [9]Average values from the 4 Storage times; [10]Values averaged from all replicas. storage times and genogroups.
