# Supplementary material for: Design and Evaluation of a Macroarray for Detection, Identification, and Typing of Viral Hemorrhagic Septicemia Virus (VHSV)
Source: Animals (Basel). 2021 Mar 16;11(3):841. doi: 10.3390/ani11030841 (PMC8002285; doi:10.3390/ani11030841)
Supplement: Supplementary file 1 [file animals-11-00841-s001.zip › Sppl Files/Sppl Table PDF/Supplementary Table S1.pdf]

Supplementary Table S1.- Data of RT-qPCR and regression lines for all replicas and repeats

[1]G1: FR-07-21

[2]Dil.	[3]0 h						1d						1 w						3 m						[9]Reproducibility		
	[4]Rpl 1	Rpl 2	Rpl 3	[5]Avrg.	[6]Desv	[7]CV	Rpl 1	Rpl 2	Rpl 3	Avrg.	Desv	CV	Rpl 1	Rpl 2	Rpl 3	Avrg.	Desv	CV	Rpl 1	Rpl 2	Rpl 3	Avrg.	Desv	CV	Avrg.	Desv	CV
-1	23.47	22.21	22.40	22.69	0.68	2.99	22.47	22.21	22.00	22.23	0.24	1.06	22.43	24.30	22.71	23.15	1.01	4.36	22.91	22.86	23.13	22.97	0.14	0.63	22.76	0.64	2.83
-2	25.26	26.10	25.19	25.52	0.51	1.98	25.26	25.10	25.19	25.18	0.08	0.32	26.50	26.11	25.70	26.10	0.40	1.53	26.67	26.63	26.58	26.63	0.05	0.17	25.86	0.64	2.48
-3	29.56	30.12	29.01	29.56	0.56	1.88	29.56	29.12	29.00	29.23	0.29	1.01	29.85	29.93	29.62	29.80	0.16	0.54	29.41	29.36	29.24	29.34	0.09	0.30	29.48	0.36	1.23
-4	32.98	33.13	32.69	32.93	0.22	0.68	32.98	32.13	32.69	32.60	0.43	1.33	32.09	32.33	32.43	32.28	0.17	0.54	34.04	33.74	33.88	33.89	0.15	0.44	32.93	0.67	2.03
-5	35.93	36.56	35.45	35.98	0.56	1.55	35.93	35.56	35.45	35.65	0.25	0.71	35.88	37.16	36.05	36.36	0.70	1.91	36.92	37.01	36.85	36.93	0.08	0.22	36.23	0.64	1.75
-6	38.76	39.16	38.56	38.83	0.31	0.79	38.85	38.76	38.25	38.62	0.32	0.84	38.93	38.93	39.07	38.98	0.08	0.21	39.89	40.12	39.31	39.77	0.42	1.05	39.05	0.53	1.35
-7	40.97	-	-	-	-	-	-	-	-	-	-	-	-	41.08	-	-	-	-	-	-	-	-	-	-	-	-	-
Rpl 1	y = 3.1966x + 19.805			R^2 = 0.99252			y = 3.3523x + 19.109			R^2 = 0.99586			y = 3.2251x + 19.659			R^2 = 0.99529			y = 3.4366x + 19.612			R^2 = 0.99561					
Rpl 2	y = 3.4040x + 19.299			R^2 = 0.99492			y = 3.3469x + 18.766			R^2 = 0.99873			y = 3.1057x + 20.590			R^2 = 0.98551			y = 3.4806x + 19.438			R^2 = 0.99746					
Rpl 3	y = 3.2931x + 19.024			R^2 = 0.99743			y = 3.3063x + 18.858			R^2 = 0.99622			y = 3.3046x + 19.364			R^2 = 0.99865			y = 3.3243x + 19.863			R^2 = 0.99351					
Avrg.	y = 3.2979x + 19.376			R^2 = 0.99710			y = 3.3351x + 18.911			R^2 = 0.99759			y = 3.2118x + 19.871			R^2 = 0.99707			y = 3.4138x + 19.638			R^2 = 0.99585					

G2: DK-1p49

Dil.	0 h						1d						1 w						3 m						Reproducibility		
	Rpl 1	Rpl 2	Rpl 3	Avrg.	Desv	CV	Rpl 1	Rpl 2	Rpl 3	Avrg.	Desv	CV	Rpl 1	Rpl 2	Rpl 3	Avrg.	Desv	CV	Rpl 1	Rpl 2	Rpl 3	Avrg.	Desv	CV	Avrg.	Desv	CV
-1	22.95	23.09	22.75	22.93	0.17	0.75	22.56	23.02	23.55	23.04	0.50	2.15	22.85	22.72	23.25	22.94	0.28	1.20	22.95	23.09	22.75	22.93	0.17	0.75	22.96	0.27	1.17
-2	26.32	25.71	26.13	26.05	0.31	1.20	27.33	27.14	27.67	27.38	0.27	0.98	27.20	28.70	27.74	27.88	0.76	2.73	26.32	27.71	27.13	27.05	0.70	2.58	27.09	0.84	3.11
-3	29.03	30.56	29.92	29.84	0.77	2.58	30.70	31.57	31.72	31.33	0.55	1.76	29.03	30.06	28.92	29.34	0.63	2.14	30.03	30.56	29.92	30.17	0.34	1.13	30.17	0.92	3.04
-4	33.74	33.31	32.22	33.09	0.78	2.37	33.49	34.92	33.46	33.96	0.83	2.46	33.74	33.31	33.22	33.42	0.28	0.83	33.86	33.34	34.22	33.81	0.44	1.31	33.57	0.64	1.91
-5	35.62	36.86	35.32	35.93	0.82	2.27	35.83	35.67	35.36	35.62	0.24	0.67	35.62	35.74	35.27	35.54	0.24	0.69	36.62	36.86	36.32	36.60	0.27	0.74	35.92	0.59	1.64
-6	39.27	39.20	39.25	39.24	0.04	0.09	40.64	39.58	39.98	40.07	0.54	1.34	39.76	38.37	39.59	39.24	0.76	1.93	39.27	39.85	40.02	39.71	0.39	0.99	39.57	0.56	1.42
-7	-	-	-	-	-	-	-	-	-	-	-	-	-	41.0	-	-	-	-	-	-	-	-	-	-	-	-	-
Rpl 1	y = 3.2631x + 19.734			R^2 = 0.99283			y = 3.3911x + 19.889			R^2 = 0.98747			y = 3.272x + 19.915			R^2 = 0.98854			y = 3.3237x + 19.875			R^2 = 0.99522					
Rpl 2	y = 3.3357x + 19.780			R^2 = 0.99136			y = 3.1926x + 20.809			R^2 = 0.96995			y = 2.9320x + 21.221			R^2 = 0.96777			y = 3.2580x + 20.499			R^2 = 0.99416					
Rpl 3	y = 3.2106x + 19.695			R^2 = 0.99589			y = 3.0560x + 21.261			R^2 = 0.97678			y = 3.1026x + 20.473			R^2 = 0.98335			y = 3.3777x + 19.905			R^2 = 0.99298					
Avrg.	y = 3.2698x + 19.736			R^2 = 0.99866			y = 3.2132x + 20.653			R^2 = 0.98221			y = 3.1022x + 20.536			R^2 = 0.98518			y = 3.3198x + 20.093			R^2 = 0.99671					

G3: MLA88

Dil.	0 h						1d						1 w						3 m						Reproducibility		
	Rpl 1	Rpl 2	Rpl 3	Avrg.	Desv	CV	Rpl 1	Rpl 2	Rpl 3	Avrg.	Desv	CV	Rpl 1	Rpl 2	Rpl 3	Avrg.	Desv	CV	Rpl 1	Rpl 2	Rpl 3	Avrg.	Desv	CV	Avrg.	Desv	CV
-1	23.28	23.92	21.98	23.06	0.99	4.29	23.50	22.90	23.22	23.21	0.30	1.29	23.92	21.98	23.50	23.13	1.02	4.41	23.28	23.92	22.90	23.37	0.52	2.21	23.19	0.67	2.88
-2	25.92	26.63	25.38	25.98	0.63	2.41	26.48	26.14	27.59	26.74	0.76	2.84	26.63	25.38	29.48	27.16	2.10	7.74	25.92	26.63	26.14	26.23	0.36	1.39	26.53	1.11	4.18
-3	29.99	30.54	30.16	30.23	0.28	0.93	31.89	31.73	30.36	31.33	0.84	2.68	30.54	29.56	31.89	30.66	1.17	3.82	29.99	30.54	31.73	30.75	0.89	2.89	30.74	0.84	2.73
-4	33.81	34.80	33.67	34.09	0.62	1.81	32.58	32.26	35.20	33.35	1.61	4.84	34.80	35.67	34.58	35.02	0.58	1.65	33.81	34.80	34.26	34.29	0.50	1.45	34.19	1.02	2.97
-5	35.93	35.41	35.96	35.77	0.31	0.86	36.59	36.55	36.94	36.69	0.21	0.58	37.41	36.96	36.59	36.99	0.41	1.11	36.93	37.41	38.55	37.63	0.83	2.21	36.77	0.82	2.23
-6	39.65	39.25	39.76	39.55	0.27	0.68	39.82	39.41	41.18	40.14	0.93	2.31	39.85	38.52	39.45	39.27	0.68	1.74	40.12	39.98	39.20	39.77	0.50	1.25	39.68	0.64	1.61
-7	-	-	-	-	-	-	-	-	-	-	-	-	-	41.15	-	-	-	-	-	-	-	-	-	-	-	-	-

	0 h		1d		1 w		3 m	
Rpl 1	y = 3.3057x + 19.860	R^2 = 0.99426	y = 3.2177x + 20.548	R^2 = 0.97902	y = 3.3214x + 20.567	R^2 = 0.99124	y = 3.4586x + 19.570	R^2 = 0.99716
Rpl 2	y = 3.0643x + 21.033	R^2 = 0.97849	y = 3.2660x + 20.067	R^2 = 0.97533	y = 3.5300x + 18.990	R^2 = 0.95565	y = 3.3400x + 20.523	R^2 = 0.99214
Rpl 3	y = 3.5471x + 18.737	R^2 = 0.99193	y = 3.5054x + 20.146	R^2 = 0.99047	y = 2.9649x + 22.205	R^2 = 0.96259	y = 3.4646x + 20.004	R^2 = 0.96813
Avrg.	y = 3.3057x + 19.877	R^2 = 0.99023	y = 3.3297x + 20.254	R^2 = 0.99257	y = 3.2721x + 20.587	R^2 = 0.98274	y = 3.4210x + 20.032	R^2 = 0.99114

GIVa: US-Makah

Dil.	0 h						1d						1 w						3 m						Reproducibility		
	Rpl 1	Rpl 2	Rpl 3	Avrg.	Desv	CV	Rpl 1	Rpl 2	Rpl 3	Avrg.	Desv	CV	Rpl 1	Rpl 2	Rpl 3	Avrg.	Desv	CV	Rpl 1	Rpl 2	Rpl 3	Avrg.	Desv	CV	Avrg.	Desv	CV
-1	21.96	22.73	22.77	22.49	0.46	2.03	22.78	22.79	22.91	22.83	0.07	0.32	22.37	22.88	22.56	22.60	0.26	1.14	23.73	24.88	23.78	24.13	0.65	2.69	23.01	0.77	3.36
-2	25.03	26.37	26.63	26.01	0.86	3.30	26.07	26.77	25.87	26.24	0.47	1.80	25.17	25.48	24.99	25.21	0.25	0.98	26.58	26.77	27.07	26.81	0.25	0.92	26.07	0.74	2.85
-3	29.48	29.84	29.48	29.60	0.21	0.70	29.18	29.77	30.41	29.79	0.62	2.07	28.87	28.96	29.54	29.12	0.36	1.25	29.84	31.77	31.18	30.93	0.99	3.20	29.86	0.87	2.92
-4	33.27	33.28	33.29	33.28	0.01	0.03	33.47	32.84	34.44	33.58	0.81	2.40	32.56	31.95	33.39	32.63	0.72	2.21	33.28	32.84	33.47	33.20	0.32	0.97	33.17	0.60	1.81
-5	36.54	36.85	36.63	36.67	0.16	0.43	36.73	35.71	34.92	35.79	0.91	2.54	35.68	34.92	33.88	34.83	0.90	2.59	36.85	36.71	37.73	37.10	0.55	1.49	36.10	1.09	3.02
-6	39.53	38.36	39.68	39.19	0.72	1.84	39.64	39.42	39.86	39.64	0.22	0.55	40.56	40.02	40.86	40.48	0.43	1.05	39.50	40.23	40.65	40.13	0.58	1.45	39.86	0.68	1.70
-7	-	40.16	-	-	-	-	-	-	-	-	-	-	-	-	-	-	-	-	-	-	-	-	-	-	-	-	-

	0 h		1d		1 w		3 m	
Rpl 1	y = 3.6049x + 18.351[8]	R^2 = 0.99623	y = 3.4449x + 19.255	R^2 = 0.99754	y = 3.6049x + 18.251	R^2 = 0.99468	y = 3.2314x + 20.320	R^2 = 0.99843
Rpl 2	y = 3.2294x + 19.935	R^2 = 0.98962	y = 3.2297x + 19.913	R^2 = 0.99754	y = 3.3431x + 19.001	R^2 = 0.98952	y = 3.0754x + 21.436	R^2 = 0.98241
Rpl 3	y = 3.3817x + 19.577	R^2 = 0.99866	y = 3.3123x + 19.809	R^2 = 0.97537	y = 3.4863x + 18.668	R^2 = 0.96414	y = 3.3891x + 20.451	R^2 = 0.99622
Avrg.	y = 3.4053x + 19.288	R^2 = 0.99702	y = 3.3290x + 19.659	R^2 = 0.99645	y = 3.4781x + 18.640	R^2 = 0.98702	y = 3.2320x + 20.736	R^2 = 0.99625

GIVb: Goby 1F

Dil.	0 h						1d						1 w						3 m						Reproducibility		
	Rpl 1	Rpl 2	Rpl 3	Avrg.	Desv	CV	Rpl 1	Rpl 2	Rpl 3	Avrg.	Desv	CV	Rpl 1	Rpl 2	Rpl 3	Avrg.	Desv	CV	Rpl 1	Rpl 2	Rpl 3	Avrg.	Desv	CV	Avrg.	Desv	CV
-1	22.86	22.73	23.77	23.12	0.57	2.45	22.85	24.88	22.47	23.40	1.30	5.54	22.78	22.39	23.91	23.03	0.79	3.43	22.85	22.72	23.25	22.94	0.28	1.20	23.12	0.72	3.13
-2	26.63	26.77	26.63	26.68	0.08	0.30	26.65	26.35	27.55	26.85	0.62	2.33	26.07	26.77	25.87	26.24	0.47	1.80	27.20	28.40	27.74	27.78	0.60	2.16	26.89	0.72	2.69
-3	30.48	29.84	30.48	30.27	0.37	1.22	30.08	29.81	29.54	29.81	0.27	0.91	29.18	29.77	29.41	29.45	0.30	1.01	30.70	31.57	31.72	31.33	0.55	1.76	30.22	0.81	2.67
-4	32.74	33.28	32.29	32.77	0.50	1.51	33.52	32.94	32.51	32.99	0.51	1.54	33.47	32.84	33.44	33.25	0.36	1.07	33.74	34.92	33.46	34.04	0.77	2.28	33.26	0.69	2.07
-5	35.91	36.85	36.63	36.46	0.49	1.35	36.45	35.12	36.92	36.16	0.93	2.58	36.85	35.74	35.92	36.17	0.60	1.65	35.62	35.81	35.27	35.57	0.27	0.77	36.09	0.63	1.74
-6	38.53	38.36	39.68	38.86	0.72	1.85	38.91	38.71	39.73	39.12	0.54	1.38	39.74	39.28	39.29	39.44	0.26	0.67	39.96	39.84	40.48	40.09	0.34	0.85	39.38	0.64	1.63
-7	40.57	41.21	-	-	-	-	41.19	40.87	-	-	-	-	-	-	-	-	-	-	-	-	-	-	-	-	-	-	-

	0 h		1d		1 w		3 m	
Rpl 1	y = 3.0986x + 20.347	R^2 = 0.99277	y = 3.2326x + 20.096	R^2 = 0.9949	y = 3.4694x + 19.205	R^2 = 0.99753	y = 3.2529x + 20.293	R^2 = 0.98849
Rpl 2	y = 3.1951x + 20.122	R^2 = 0.98889	y = 2.8169x + 21.443	R^2 = 0.9909	y = 3.2694x + 19.689	R^2 = 0.99582	y = 3.1766x + 21.092	R^2 = 0.96228
Rpl 3	y = 3.1817x + 20.444	R^2 = 0.99345	y = 3.3537x + 19.715	R^2 = 0.9886	y = 3.1737x + 20.199	R^2 = 0.99344	y = 3.1566x + 20.939	R^2 = 0.97129
Avrg.	y = 3.1585x + 20.304	R^2 = 0.99612	y = 3.1344x + 20.418	R^2 = 0.9996	y = 3.3042x + 19.698	R^2 = 0.9992	y = 3.1668x + 20.930	R^2 = 0.97486

Total Reproducibility[10]		
Avrg.	Desv	CV
23.01	0.64	2.77
26.49	0.93	3.51
30.09	0.87	2.88
33.42	0.84	2.50
36.22	0.80	2.22

39.51	0.65	1.66

[1]Genogroup and reference strain; [2]Dilution; [3]Storage time; [4]Replica; [5]Average Ct; [6]Standard deviation; [7]Coefficient of variation; [8]Standar curve and coefficient of determination; [9]Average values from the 4 Storage times; [10]Values averaged from all replicas. storage times and genogroups.
